# Is your curriculum GenAI-proof? A method for GenAI impact assessment and a case study

**DOI:** 10.12688/mep.20815.1

**Published:** 2025-03-26

**Authors:** Remco Jongkind, Erik Elings, Erik Joukes, Tom Broens, Hemmik Leopold, Floris Wiesman, Jennita Meinema

**Affiliations:** 1 Teaching and Learning Centre, Amsterdam UMC Location AMC, Amsterdam, North Holland, 1105AZ, The Netherlands; 2 Medical Informatics, Amsterdam UMC Location AMC, Amsterdam, North Holland, 1105AZ, The Netherlands

**Keywords:** Generative AI, GenAI, Large Language Models, Curriculum adaptation, ChatGPT, assessment validity, AI literacy, learning outcome relevance.

## Abstract

**Background:**

Generative AI (GenAI) such as ChatGPT can take over tasks that previously could only be done by humans. Although GenAI provides many educational opportunities, it also poses risks such as invalid assessments and irrelevant learning outcomes.

This article presents a broadly applicable method to (1) determine current assessment validity, (2) assess which learning outcomes are impacted by student GenAI use and (3) decide whether to alter assessment formats and/or learning outcomes. This is exemplified by the case-study on our medical informatics curriculum.

We developed a five-step method to evaluate and address the impact of GenAI. In a collaborative manner, the courses in a curriculum are analysed on their assessment plans and together with the teachers, the courses are adapted to address the impact of GenAI usage.

**Results:**

57% of assessments, especially in writing and programming, were at risk of reduced validity and relevance. GenAI impact on assessment validity was closer related to the content and structure of assessments than their complexity according to Bloom’s taxonomy. During educational retreats, lecturers discussed the relevance of impacted learning outcomes and whether students should be able to achieve them with or without GenAI.

Furthermore, the results led to a plan to increase GenAI literacy and use over the years of study. Subsequently the coordinators were asked to either adjust either their assessments formats to preclude GenAI use, or to alter the learning outcomes and include GenAI use and literacy. For 64% of the impacted assessments the assessment format was adapted and for 36% the learning outcomes were adapted.

**Conclusion:**

The majority of assessments in our curriculum were at risk of reduced assessment validity and relevance of learning outcomes, leading us to adapt either the assessments or learning outcomes. This method and case-study offer a potential blueprint for educational institutions facing similar challenges.

## List of abbreviations

GenAI: Generative Artificial Intelligence

LLM: Large Language Models

MI - Medical Informatics

UvA - University of Amsterdam

NVMO – Nederlandse Vereniging voor Medisch Onderwijs - Dutch Society for Medical Education

GPT - Generative Pre-trained Transformer (specific to ChatGPT)

## Introduction

In recent years, the application of artificial intelligence (AI) in various domains has shown great potential to perform certain tasks faster and/or better than human professionals. Initially, AI was mainly used for data, sound and/or image analysis and the impact was therefore limited to certain professions and tasks. However, with the introduction of ChatGPT 3.5 in November 2022, it became clear that new forms of generative artificial intelligence (GenAI), such as large language models (LLM), may also be able to take over and assist with tasks in other professions such as medical informaticians or doctors, which were previously thought to be exclusively human tasks. GenAI refers to AI systems that produce new content based on input data, known as prompts. These systems can create text, images, audio, and other forms of digital content. This article focuses primarily on large language models, and particularly the most used variant ChatGPT, a type of GenAI designed to generate coherent and contextually relevant text based on user prompts.

There are many examples that show that GenAI can take over and aid human processes in the working field. For instance, Ayers *et al.* (2023) showed that ChatGPT v3.5 answered patients' questions in an online forum more empathetically and more correctly than doctors. ChatGPT v4, an improved version of ChatGPT v3.5, can effectively design, write and debug software code ( Azaiz *et al.*, 2023; Ellis *et al.*, 2024; Górecki, 2024; Idrisov & Schlippe, 2024; Kabir *et al.*, 2024; Kwon *et al.*, 2023; Palkowski & Gruzewski, 2024; Popovici, 2023; Reason *et al.*, 2024; Rodriguez *et al.*, 2024; Song *et al.*, 2024; Su & McMillan, 2024; Yeo *et al.*, 2024). ChatGPT can also be used in health economics modelling ( Reason *et al.*, 2024) and reviewing mobile health apps ( Giunti & Doherty, 2024).

In the university education domain, GenAI is poised to transform teaching and assessment practices, raising new questions about its potential and challenges. ChatGPT and other GenAI can be used as a tool for many aspects of writing, such as writing abstracts ( Cheng *et al.*, 2023; Hwang *et al.*, 2024), introductions ( Sikander *et al.*, 2023) medical reports ( Zhou, 2023), case reports ( Buholayka *et al.*, 2023), scientific reviews ( Kacena *et al.*, 2024; Nazzal *et al.*, 2024), cover letters ( Deveci *et al.*, 2023), patient educational material ( Mondal *et al.*, 2023), essays ( Herbold *et al.*, 2023), research articles ( Abuyaman, 2023; Huespe *et al.*, 2023; Lund & Ting, 2023; Saito & Tsukiyama, 2024; Semrl *et al.*, 2023) and peer reviews ( Saad *et al.*, 2024). However, its performance in referencing is subpar, and it is currently not particularly suitable as a tool for finding and using references ( Lechien *et al.*, 2024; Mugaanyi *et al.*, 2024). However, considering the rapidly evolving capabilities of GenAI, in the near future referencing might also possible ( Dell’Acqua *et al.*, 2023).

ChatGPT or other GenAI have demonstrated the ability to pass many different types of exams in varying domains. For example it has passed graduate biomedical, physiology and biochemistry exams ( Ghosh & Bir, 2023; Stribling *et al.*, 2024; Subramani *et al.*, 2023), clinical informatics ( Kumah-Crystal *et al.*, 2023), graduate Python programming ( Ellis *et al.*, 2024), and a wide range of entry and specialist medical exams in a wide range of countries such as Germany, the United States and the United Kingdom ( Cuthbert & Simpson, 2023; Farhat *et al.*, 2024; Griewing *et al.*, 2023; Kufel *et al.*, 2024; Li *et al.*, 2024; Lum, 2023; Meyer *et al.*, 2024; Sarangi *et al.*, 2024; Stengel *et al.*, 2024). 

Previous examples show that GenAI has abilities that overlap with learning outcomes that are part of many curricula. Especially skills in knowledge reproduction, scientific writing, programming, data analysis and communication seem to be impacted. This is also the case for our Medical Informatics curriculum in which students learn to understand the basic medical subjects, the methodology of medical-scientific research, software development and the organization of healthcare. As GenAI advances, its expanding role in medical informatics is likely to create both new opportunities and significant challenges for professionals in the field.

Many students currently use ChatGPT and other GenAI in their studies, both at our university and at others ( De Winter *et al.*, 2023). We conducted an informal anonymous poll amongst ~35 of our medical informatics bachelor students and 95% indicated that they used ChatGPT in some form for their assessments. Another informal poll amongst ~100 of our third-year medical students gave a similar percentage (90%). This could be because some students tend to choose the path of least resistance ( Abbas *et al.*, 2024). Uninformed use of GenAI is risky since students are not always aware of potential biases and incorrect answers that GenAI might provide. Furthermore, by using GenAI for assessments students might not achieve the learning outcomes or learn the outcomes that are relevant to the work field. 

Despite its potential, universities remain cautious about integrating GenAI into their curricula, citing concerns over academic integrity, assessment validity, and privacy. Additionally, the need to establish GenAI infrastructures that are accessible for a diverse population remains a significant challenge. However, universities are gradually beginning to explore pilot programs with GenAI. For instance, the University of Amsterdam (UvA) recently released policy guidelines stating that students may use GenAI if explicitly permitted at the course level. Similarly, other universities have introduced policies that stress students' responsibility for ensuring transparency and accountability in their work.

In mid-2023, some of our more proactive and tech-savvy lecturers began adjusting their assessments and teaching methods to incorporate these new developments in GenAI. However, this posed the risk of disrupting curriculum cohesion, as not all courses adopted GenAI usage. For example, students might initially learn a skill using GenAI in one course, only to face restrictions on its use in a later course, making it more difficult or impossible for them to complete the assessment.

Therefore, we recognized the importance of assessing the impact of GenAI on assessments and learning outcomes across the entire curriculum. Our goal was to make any necessary adjustments to ensure that assessments remain valid, learning outcomes stay relevant to future professional demands, and there is a consistent approach to GenAI use and policy. Ultimately, our goal is safeguarding the quality of our students' learning experiences.

At the time there was some initial reporting on the impact of GenAI on the assessment or course level ( Chaudhry *et al.*, 2023). However, a comprehensive approach to evaluating its impact across an entire curriculum, with a consistent method for all assessments and courses, was lacking. The only curriculum-wide initiative we found was a methodology being developed by van Ooijen-van der Linden *et al.* (2023) to assess the impact of all forms of AI, not just GenAI. However, their methodology was still in early stages and not yet ready for broader implementation. Additionally, it relied on time-consuming series of group discussions among teachers, staff, and students, which we anticipated would not garner sufficient support from our teaching faculty. Informal inquiries within the Dutch Society for Medical Education (NVMO) working group on academic development and the Teaching and Learning Centres at other University of Amsterdam faculties also failed to yield suitable methodologies. Consequently, we decided to develop our own method to assess the curriculum wide impact of GenAI on assessment validity and relevance.

Our hypothesis in March 2023 was that the assessments involving writing of non-academic products at home would be most impacted. Followed by writing academic products and software coding at home. We also expected that due to its hallucinations ChatGPT would perform worse at the “lower” Bloom levels of remembering and understanding. We anticipated that ChatGPT would perform at its best at intermediate Bloom levels of applying and analysing and would drop off again at the higher levels of creating and evaluating. Therefore, we expected that GenAI would be able to make more assessments in year 1 compared to year 3 due to the increase in (Bloom) levels of learning outcomes over the years in our curriculum. 

This article outlines the method and the results of the AI impact scan conducted on the Medical Informatics BSc. curriculum as a case study. It also details the steps we took to future-proof the curriculum, along with practical tips and recommendations for other educational programs based on our experiences.

## GenAI impact scan method – determining the impact on assessment validity and relevance of learning outcomes

In March 2023, five months after the launch of ChatGPT3.5, we started to develop and subsequently implement a five-step method to assess the impact of GenAI at a curriculum level and subsequently adapt the curriculum accordingly to make sure the constructive alignment stays intact. Our method aimed to identify which types of assessments and learning objectives are most impacted by student use of GenAI.

We developed a multi-step approach to address this issue ( Table 1). First, we determined the urgency with which action was needed (Step 1). This because it was unclear to us what the extent of the impact would be and to create awareness and willingness for change among management and teachers. Next, we excluded assessments where students had no opportunity to use GenAI tools (Step 2). This method incrementally funnels the assessments on their risk of being invalidated by GenAI. This also reduces the amount of assessments in focus and hence the workload in later steps. Afterward, we assessed the potential impact by consulting the lecturers, who serve as subject matter experts (Step 3). To ensure coherent curriculum adjustments, we conducted a collaborative discussion with the lecturers (Step 4). Finally, given the rapid evolution of GenAI capabilities ( Dell’Acqua *et al.*, 2023), we plan to repeat Steps 2, 3 and 4 annually for each assessment.

**Table 1.** Table describing the five steps of the GenAI impact scan method with the goal, the input needed for the step, the outcomes of the step and the involved actors.

	Goal	Input	Outcomes	Actors
**Step 1**	Estimating the extent to which learning activities are impacted to determine urgency	Set of learning activities used in the curriculum,	Set of used learning activities that are impacted by GenAI	Educational advisers
**Step 2**	Determining whether students have the opportunity to make the assessment with GenAI. This reduces the number of assessments that lecturers need to screen.	Set of assessments in the curriculum	For each assessment a score that expresses the possibility of GenAI abuse. (No risk / Partial risk / High risk).	Educational advisers
**Step 3**	Determining to which degree GenAI can make the assessments. This is to decide which assessments/learning outcomes to alter.	Set of (partially) at risk assessments in the curriculum	For each partial or high-risk assessment a score that expresses the degree of ability of GenAI to successfully complete the assessment.	Lecturers & educational advisors
**Step 4**	Determining if/how to adapt the assessments or learning outcomes	Set of assessments risk scores per assessment, set of affected learning outcomes of all courses. Exit competences were not considered due to their general nature.	Decision per learning goal whether to keep, adapt or remove it. List of new learning outcomes that we wish to add to the curriculum, for example AI literacy.	Lecturers, educational advisers, educational management
**Step 5**	Ensuring the ongoing validity and relevance of assessments and learning outcomes by repeating step 2 and 3 yearly in the PDCA cycle.	The assessments per course	For each assessment an indication whether it is at risk and a score that expresses the degree of ability of GenAI to successfully complete the assessment.	Lecturers

### Step 1: Pre-scan

In the first step characteristic assessment formats are evaluated at a curriculum level and it is estimated to which degree GenAI can be used to make these assessments. This is done to give an initial impression of the impact that GenAI has and to decide to which degree the educational management had to prioritize the AI impact scan. Examples of such activities include team-based learning, project-based education, and workplace learning.

We expect project- and case-based reports, programming take-home exams and academic writing to be aspects most impacted. This since many of these assessments occur without direct supervision and generally involve actions that GenAI is proficient in. For programming and (academic) writing, these are activities that many studies have already shown to be heavily impacted using GenAI as discussed in depth in the introduction.

### Step 2: Quick scan

In the second step educational advisors determine which assessments are at risk because students have the opportunity to make the summative assessments unnoticed with GenAI and therefore not reach the learning outcomes. 

In this step the educational advisors go through all summative assessment of the curriculum and answer the following 3 questions for each assessment:

1. Is the assessment conducted under supervision?
  When an assessment is done under supervision, the likelihood of students displaying academic dishonesty is much lower ( Janke *et al.*, 2021; Yazici *et al.*, 2011)
2. Are there intermediate products for the final assessment?
  Intermediate products have been employed by many leading universities such as Caltech University, Cornell University and University of Tokio to improve the authenticity and validity of the assessments ( Moorhouse *et al.*, 2023).
3. Is the weight of the assessment <10% of the total of the course?
  This question was mainly asked to prioritize which assessments to analyse and if necessary, to adapt first.

If the answers to all three above questions are no, then the assessment is classified as *high-risk*. If the answer to question 1 is no, then the assessment is classified as *partial risk*. Otherwise, the assessment is classified as *no risk*. For all high or partial risk assessments a detailed scan is performed with priority being given to the high-risk assessments.

### Step 3: Detailed scan

In the third step lecturers responsible for the assessments determine whether the assessments that are deemed at risk can in fact be made with GenAI.

In this step, two hands-on workshops are organized for the course coordinators and lecturers in which they are taught the basics about GenAI and how to effectively prompt. For this workshop the coordinators and lecturers should be provided with GenAI access. Ideally a tool is provided which mirrors what students are likely to use, uses an advanced frontier large language model and has functionalities such as uploading files, creating images, using plugins, custom GPTs (or similar) and no training on input data.

In these workshops, lecturers make their own at-risk assessments with GenAI. If they do not manage all their assessments during the workshops they are asked to continue later and report back via a central form within a stated period.

The lecturers are asked to classify their assessments on a Likert scale of 1–5 with the labels below created by the educational advisors. These labels were created by the educational advisors and validated on one first-year bachelor's course and one first-year master's in medical informatics. These courses were chosen as representative for the curriculum by the educational management. Based on the initial validation minor adjustments were made to the levels.

Rating scale:

1. ChatGPT gives a perfect or very good solution for the assessment. The prompt given to ChatGPT was (almost) literally the assessment description.
2. ChatGPT gives a perfect or very good solution for the assessment. The prompt given to ChatGPT had to be refined based on the assessment description. This requires knowledge of how to prompt effectively.
3. ChatGPT gives an insufficient solution based on just the assessment and good prompts. Additional subject matter knowledge or skills are necessary for a perfect or good solution.
4. ChatGPT gives an insufficient solution for the assessment, even with good prompting and additional knowledge subject matter knowledge or skills. ChatGPT can only be used for inspiration, for example to brainstorm.
5. It is not possible to use ChatGPT for this assessment. For example, because actions must be taken in certain software systems.

The rating scale provides a measure of the resistance a student may encounter for using GenAI for an assessment, because students tend to choose the path of least resistance ( Abbas *et al.*, 2024). For example, for an assessment where (almost) literal copying of the assessment description results in a good outcome (level 1), the threshold for using GenAI is lower compared to an assessment that requires modifications to the prompt to achieve a good result (level 2). Therefore, we expect that each scale represents a (non-linear) increase in difficulty and utility for a student to utilize GenAI for the assessment. Furthermore, the score indicates which skills or knowledge the students still need to complete the assessment, namely GenAI literacy skills or subject matter expertise.

With all assessments in the curriculum rated, we can prioritize adjustments and decide how to adjust the assessments or learning outcomes. The educational management decided that scores 1, 2 and 3 for assessments were undesirable and that either the assessment format or the learning outcomes should be adapted. 

### Step 4: Curriculum adaptation

Based on the risk scores, clusters are made of related assessments and their associated learning outcomes.

Examples of clusters could be:

1. Academic skills
2. Programming
3. Writing skills (academic and non-academic)
4. Meta-cognitive skills
5. Data (synthesizing, analysis, etc.)

Subsequently in a meeting of ~3 hours with all lecturers the impact on the learning outcomes is discussed. The lecturers are divided into groups according to their expertise and asked to determine for each of their assigned learning outcomes whether they would like to start, adapt or continue teaching learning outcomes considering the advances in GenAI. With “start” being new learning goals to be added to the curriculum (e.g. AI literacy), “adapt” being adding GenAI use to the learning outcome and “continue”, continue teaching this skill without GenAI use. For the category “adapt” the lecturers should indicate from which year of the study GenAI use should be allowed for that learning outcome. This so that the students learn the basis without GenAI, to be able to critically evaluate the output, and later are allowed to use GenAI. This method is an adjusted format of the Start, Stop, Continue methodology from Cowan & George (1999).

Based on this meeting's input, the educational management can formulate a policy for the coherent adjustment and addition of learning outcomes to the curriculum.

Subsequently, based on this policy course coordinators can then adapt their at-risk assessments and learning outcomes according to the guidelines. These adaptations fall into one of two categories:

- Change the assessment format to prevent GenAI usage, ensuring students achieve the learning outcomes independently.
- Adjust the learning outcomes to better reflect the (desired or future) situation in the work field. For this, the learning outcomes, assessment and teaching must be adapted so that the students can achieve the new or adapted learning outcomes. For example, by learning how to make a business plan for an e-health app in co-creation with GenAI.

### Step 5: Consolidation

Since the abilities of GenAI are rapidly improving and the boundary of capabilities is increasing ( Dell’Acqua *et al.*, 2023) it is important to constantly keep evaluating the impact of GenAI use by students on assessment validity and relevance.

Therefore, the quick scan (step 2) and the detailed scan (step 3) are included in the Plan Do Check Act (PDCA) cycle of each course.

**Figure 1.**
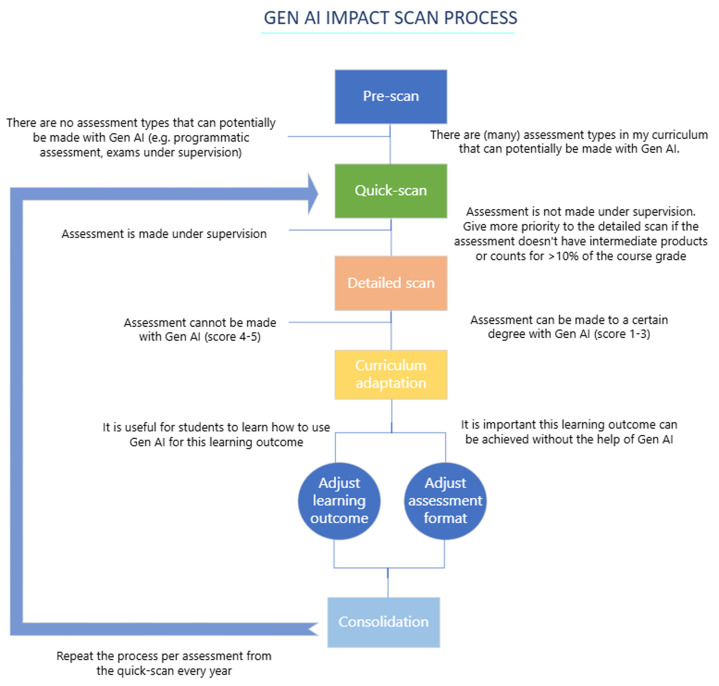
Process flow of the GenAI impact scan with per step the questions to be answered or decisions to be made.

The process and decisions to be made during the five steps as described above are visualized in the process flow in Figure 1.

## Results – a case study from the Bachelor of Medical Informatics

**Figure 2.**
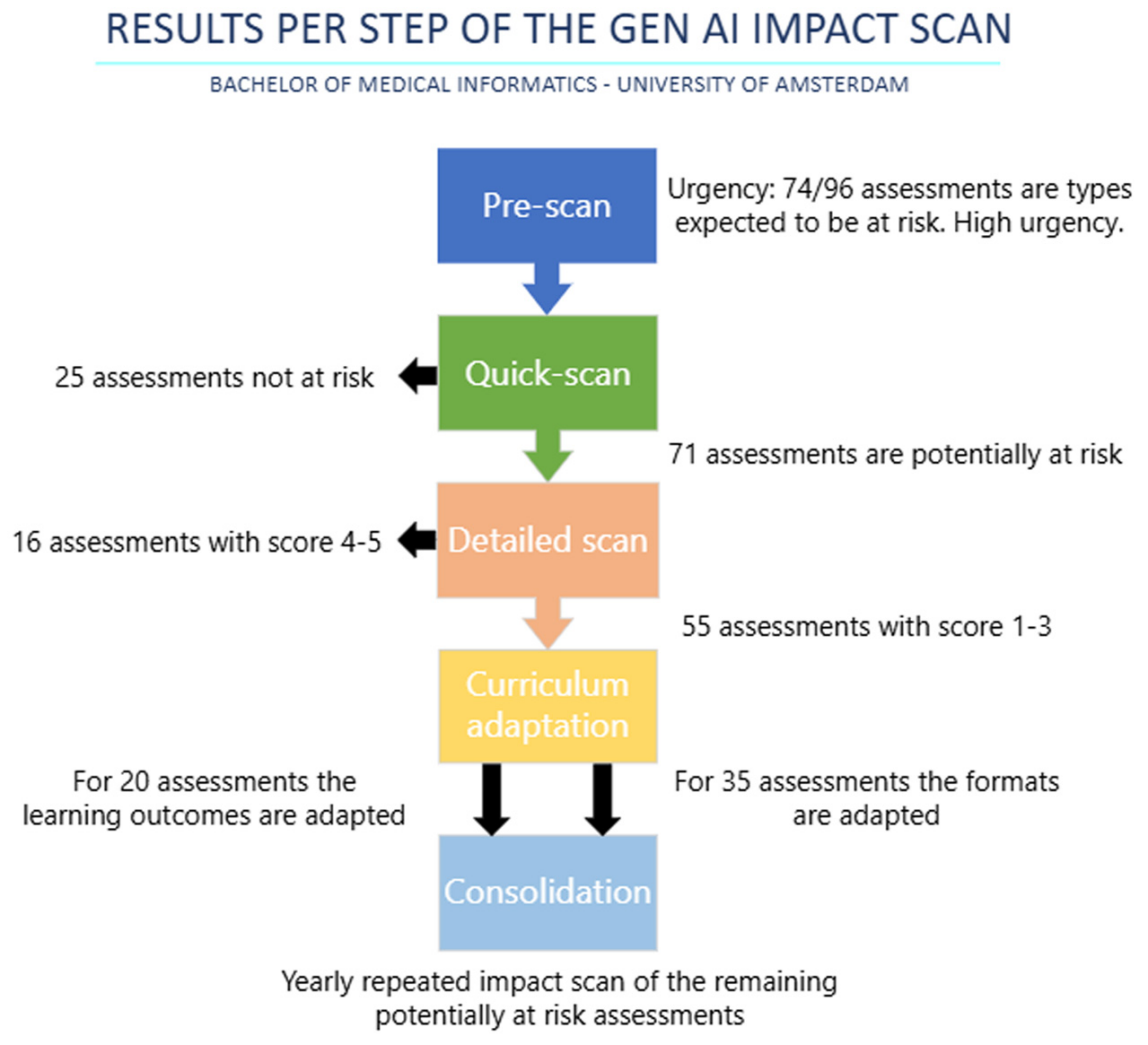
Flowchart describing the five steps of the impact scan method with the results per step.

In the below section we will highlight the case study from our own curriculum to give an indication of the impact that GenAI usage by students can have on the validity of assessments and relevance of learning outcomes of a medical informatics bachelor curriculum. The results from this case study are summarized in Figure 2. The curriculum consists of 16 courses, 169 learning outcomes and 96 assessments.

### Step 1 Pre-scan

With the pre-scan we determined what the characteristic elements of the MI curriculum were that would be impacted by GenAI. These activities were gathered from our curriculum database Act-e ( Broens *et al.*, 2017). This is a database outlining all learning goals, learning activities and assessments in our curriculum.

Subsequently we estimated to which degree these activities, or their assessments, are impacted by GenAI based on our own experiences with the capabilities with GenAI and reports from others ( Dell’Acqua *et al.*, 2023; George *et al.*, 2023; Kalla *et al.*, 2023). For example workplace learning, such as in medical residency, is relatively unaffected by GenAI as it primarily involves hands-on activities and verbal interactions, with minimal reliance on written assessments. The impact is especially low when programmatic assessment with feedback from supervisors is used.

Our bachelor uses 19 different assessment types. Given that 74 out of 96 assessments in the bachelor's program were project reports, academic writing, or programming take-home exams—types considered more vulnerable to GenAI—the next steps of the AI impact scan were considered more urgent.

### Step 2 Quick scan

After identifying the learning activities that may be impacted by GenAI during the pre-scan, it became a high priority to assess which specific assessments were at risk of being completed with GenAI. This led us to the quick scan, where we focused on identifying assessments vulnerable to unnoticed use of GenAI by students.

Educational advisors from the Teaching and Learning Centre determined which assessments were at risk because students had the opportunity to make the summative assessments unnoticed with GenAI and therefore potentially would not reach the learning outcomes. 

For our bachelor 57/96 summative assessments were at partial risk and 14/96 were high-risk. In total 71 (74%) of the assessments were (partially) at risk.

These high numbers were as expected based on the pre-scan because of the high amount of project and case-based education in the curriculum. Especially the academic reports, business plans, advice reports and take-home exams for programming were at risk.

### Step 3 Detailed scan

Having established which assessments were at risk through the quick scan, a more in-depth analysis was necessary to understand the extent of this risk. In the detailed scan, we engaged with lecturers to evaluate how and to what degree GenAI could be used to complete these assessments.

Therefore we organized 2 workshops with the lecturers, trained them in GenAI use and provided them with ChatGPT Team licences. During and following the workshop the lecturers determined which of the (partially) at risk assessments could in fact be made with GenAI. We selected ChatGPT (version 4) over other GenAI tools like Claude, Gemini, Mistral, and Llama, assuming most students would use it due to its strong brand recognition. Additionally, ChatGPT consistently ranked as the most capable tool on LLM benchmark boards, such as UC Berkeley’s LMSys Chatbot Arena, during the study period (April 2023–June 2024).

We applied scoring based on the five-point rating criteria as discussed in the method section. Fifty-five of the 71 at-risk assessments had a score of 1–3 and could thus to a certain degree be made with GenAI.

**Figure 3.**
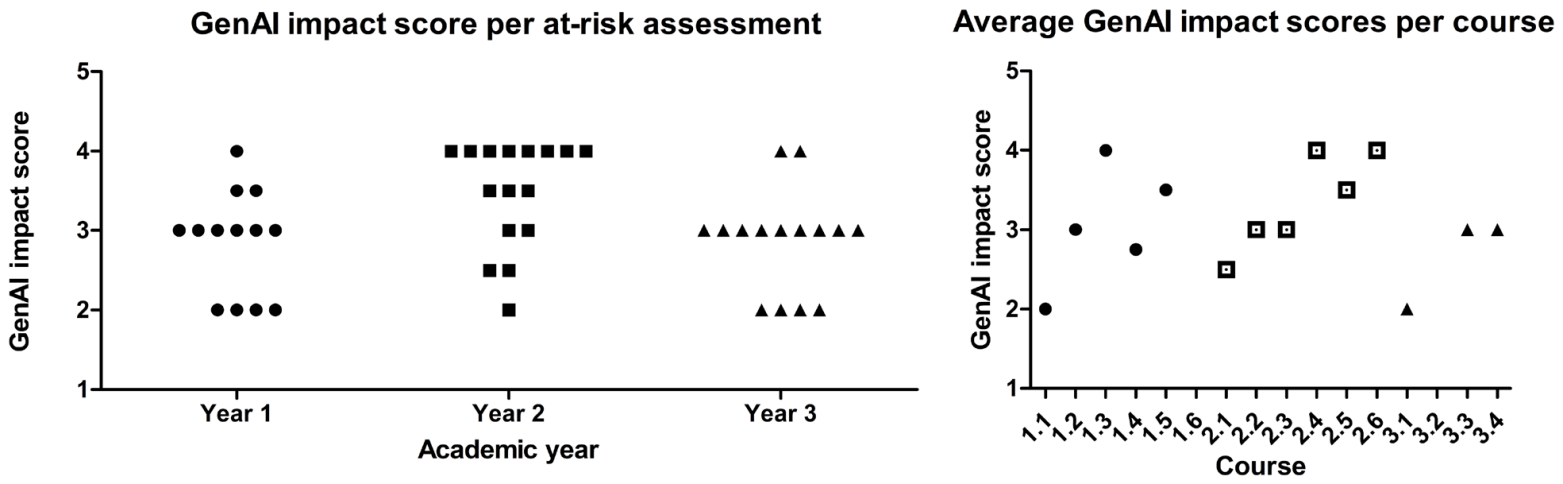
Two graphs depicting the GenAI impact scores assigned by lecturers, indicating to which degree GenAI can be used to make the assessments, per year (left) or course (right). With 1 being GenAI able to completely make the assessment and 5 that no GenAI usage is possible. For a detailed description of the interpretation of each score see step 3 in the method section. The courses where no impact score is indicated had no assessments where students have the opportunity to make the assessments with GenAI. Each dot in the left graph represents an assessment, each dot in the right graph represents an average of the assessments across a course. An assessment can have a non-integer score if some elements of the assessment fall in one score bracket and some in the other.

The AI impact score was over the entire curriculum on average 3.1/5. The average in year 1 was 2.8/5, year 2 was 3.5/5 and year 3 was 2.9/5 ( Figure 3, left). The average score of at-risk assessments per course are displayed in Figure 3 on the right. The results of these assessments were then discussed one-on-one by the curriculum committee with the coordinators of all courses in the curriculum in a general meeting.

### Step 4 Curriculum adaptation

With the detailed scan revealing which assessments could indeed be (partially) made with GenAI, it became necessary to address this identified impact. In response, we undertook a process of adapting the curriculum to mitigate the impact of GenAI on learning outcomes, resulting in a series of policy changes, assessment adjustments and learning goal adaptations.

The first step in this process was to cluster the learning outcomes into the following six categories:

1. Subject matter knowledge
2. Academic skills
3. Programming
4. Writing skills (academic and non-academic)
5. Meta-cognitive skills
6. Data (e.g., synthesizing, analysis)

Subsequently we organized an educational afternoon retreat of 3 hours with all the medical informatics lecturers. We asked them to divide into groups according to their expertise and asked each group to determine for each of their assigned learning outcomes whether they would like to start, adapt or continue teaching these learning outcomes to students considering the advances in GenAI. Each group was chaired by an educational advisor or a programme director.

Based on the input from this afternoon retreat, the curriculum committee formulated a policy on the usage of GenAI by students throughout the Bachelor of Medical Informatics.

Most learning outcomes (~80%) the lecturers indicated were important to continue teaching as they are the basics and necessary to assess the correctness of the output of GenAI. For the remaining +-20% of the learning outcomes the lecturers indicated that those could be (partially) altered to include GenAI use. Several learning outcomes were added, mainly pertaining to GenAI literacy and skills. This is to be able to effectively and responsibly use GenAI regarding privacy, data security, environmental impact, academic integrity, biases, didactic considerations such as mental complacency and output accuracy.

Subsequently the curriculum committee developed policy for the intended use of GenAI by students across the curriculum. AI is not only a technological innovation that impacts the assessment or constructive alignment of education, but it is also a tool that can positively or negatively change how students learn, and teachers teach.

In line with the policy of the University of Amsterdam, educational management recommends that first-year coordinators limit or avoid the use of GenAI to ensure students establish a solid foundation in medical informatics. During this time, students are also trained to critically evaluate GenAI output for reliability and accuracy. In the second and third years, coordinators are encouraged to explore AI use for the learning goals and it’s potential to enhance learning, while ensuring it does not fully replace traditional methods. Furthermore, it is important that if lecturers in a skill line include the use of GenAI in the learning outcomes that this is then clearly communicated to the lecturers later on to maintain constructive alignment across the curriculum. As a general principle, students are informed that the use of GenAI is not permitted unless it is explicitly stated in the course manual that it is allowed for assessments in the course.

The most common changes to the assessments are currently regarding the assessment format so that GenAI usage isn't possible anymore. Out of the 55 assessments with an impact score of 1–3, for 35 the assessment format was changed. The most common changes to assessment format were examination under supervision, changing a paper to a presentation or podcast, making the assessment process-focused and adding a number of (formatively) assessed intermediate products. This is to ensure proficiency in basic knowledge and skills. 

For the remaining 20 out of 55 assessments with a score of 1–3, mainly in programming and non-academic writing, the learning outcomes were altered or the weight in the grading rubric were adjusted. 

The most common adjustments to learning outcomes included revising rubric weightings, incorporating AI literacy, raising the difficulty of learning outcomes, and rephrasing outcomes to integrate GenAI usage.

### Step 5 Consolidation

Once the curriculum had been adapted to the GenAI impact, a process for continuous adaptation was needed since GenAI capabilities are rapidly increasing ( Dell’Acqua *et al.*, 2023). This led to the consolidation step, where a long-term strategy was developed to ensure that the curriculum remains aligned with evolving GenAI capabilities.

To ensure continuous adjustment, both the quick scan (step 2), the detailed scan (step 3) and the curriculum adaptation (step 4) are now integrated into each course’s Plan Do Check Act (PDCA) cycle. The course coordinator is asked several questions in the yearly exam evaluation report on whether students can make the assessments with GenAI and if so, what the proposed changes are. 

The answers to these questions will then also be discussed in the subsequent so-called “action plan” meeting between the course coordinator and all involved parties which precedes the start of each course. 

The upcoming academic year the steps will be executed to ensure a systematic and continuous curriculum adaptation approach. Since this will take place in the upcoming academic year, there are no results to describe at this moment. 

## Conclusion & discussion

The growing use of GenAI by students has necessitated a re-valuation and adjustment of assessments and learning outcomes of curricula. This article describes a generalizable method to determine the validity of assessments and which learning outcomes in a curriculum are impacted by the use of GenAI. It also illustrates how to decide whether to alter the assessment formats or the learning outcomes. This approach is illustrated through a case study on the medical informatics curriculum revealing that 57% of the assessments could be achieved to a certain degree using ChatGPT.

**Figure 4.**
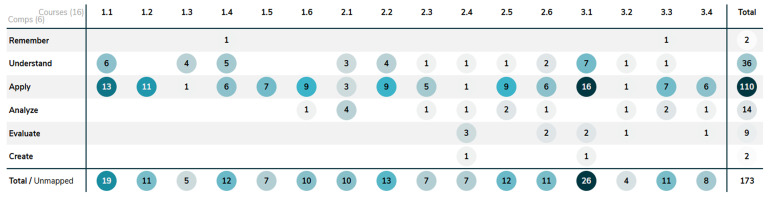
Shows the distribution of the complexity of learning outcomes, categorized by Bloom's taxonomy, across the curriculum database Act-e for courses 1.1–3.4. The first number represents the year of the course, and the second represents the order of the course within that year.

Contrary to our initial hypothesis, the usability of ChatGPT did not decrease in year 3 compared to year 1 whereas the Bloom levels of the assessments does increase ( Figure 4). The average GenAI impact score was 2.9 in year 1, 3.5 in year 2, and 2.8 in year 3. The Bloom level of the assessment does not appear to determine its performance on our GenAI impact scale but rather the content or way the assessment is formulated matters. Furthermore, the assessments were made with ChatGPT v4, which provides considerably less hallucinations than ChatGPT v3.5 and has higher performance in general (i.e. Chelli *et al.*, 2024), thus potentially improving its usability at lower Bloom levels. Since spring 2023 also other studies have been published showcasing either no difference in the performance of ChatGPT across different Bloom levels or that the performance is better at lower Bloom levels ( Ghosh & Bir, 2023; Herrmann-Werner *et al.*, 2024).

The main challenge within a curriculum is to decide which learning outcomes students should achieve without GenAI to ensure they know the basics, and which learning outcomes you want students to use with GenAI. Research shows that students with access to GenAI achieve 48 to 127% higher performance scores than students without access to GenAI. However, if in a subsequent similar task, the students that used GenAI do not have access to GenAI, they score 17% lower than students who did initially not have access to GenAI ( Bastani *et al.*, 2024). The higher performance of students using GenAI, illustrates that prohibiting the use of GenAI is not effective, since students can be expected to have to use GenAI in the workplace to be competitive ( Dell’Acqua *et al.*, 2023). Furthermore, it is important that GenAI access is consistent or increases incrementally over a curriculum, to prevent that students are suddenly no longer allowed to use GenAI for a type of learning outcome that they were allowed to use GenAI for earlier. When deciding to allow GenAI use for assessments the students need to be educated to effectively and responsibly use GenAI with regards to privacy, data security, environmental impact, academic integrity, biases, output accuracy and didactic considerations such as potential mental complacency.

Regarding our method, there are some important considerations and notes concerning the case study. First, since our main aim was to adapt the curriculum and ensure the learning of our students, a few of the assessments were already adjusted before or during our study. Therefore, the numbers provided in the case study do not represent a completely “pre-GenAI” curriculum and the impact of GenAI on the validity and relevance of assessments as portrayed in our study will be an underestimation. 

Additionally, the process by which lecturers evaluated the capability of ChatGPT to complete their assessments is subjective and dependent on the lecturers’ GenAI proficiency. To mitigate the subjectiveness, we addressed the skill dependency by providing two workshops in which we trained the lecturers in GenAI use. We also encouraged the lecturers to work together during the workshops on the assessments and discuss their verdict of the outcomes with each other and the educational advisors. Since most lecturers are medical informaticians with high digital literacy, they were well-prepared to engage with GenAI. However, when applying this method to other curricula, more extensive GenAI training may be necessary depending on the curriculum. A potential improvement could involve having student assistants attempt the assessments with GenAI to better simulate students' proficiency with both the subject matter and GenAI. This would then also address potential differences in GenAI competency between different student populations.

Beyond the impact on the learning outcomes and the (future) work field, GenAI also offers great opportunities and considerations for teachers and students during the study itself.

For instance, several studies show that GenAI tools can assist students developing writing and other skills ( Kurniati & Fithriani, 2022; Wang, 2020; Zhao, 2023). However, other studies demonstrate or predict negative effects such as the development of biases, lower ratings from assessors due to prejudices against the use of AI, the required extra attention for critical appraisal of sources, and cognitive complacency ( Abbas *et al.*, 2024; Choudhury & Shamszare, 2023; Liu *et al.*, 2022; Lund & Wang, 2023). Therefore, it is important that more research is done regarding the potential cognitive effects of GenAI use on learning, specifically on which guardrails would work best to provide optimal learning. GenAI applications that don’t instantly give away answers but encourage students to think, interact with the material, allow for reflection and provide the right amount of challenge could be a solution ( Bastani *et al.*, 2024).

To ensure students achieve the intended learning outcomes without GenAI a shift is needed from traditional written assessments to either supervised or more interactive forms of assessment, such as presentations, in-person exams, or process-focused projects that are difficult for AI to replicate ( Bower *et al.*, 2024; Chaudhry *et al.*, 2023; Kaldaras *et al.*, 2024; Zirar, 2023). A challenge with this is that these generally tend to be more time consuming for faculty and more resource intensive, needing examination or lecture rooms.

When adapting learning goals and integrating GenAI into assessments, it is important to account for the potential increase in difficulty and scope of assessments, as students using GenAI could be expected to produce more extensive output. To prevent inequity, it is essential to provide students with equal access to GenAI platforms, ensuring that those unable to afford GenAI subscriptions are not disadvantaged.

Concluding, our findings advocate for a systematic and adaptive approach to curriculum development in response to GenAI use and its impact on assessment validity and relevance. The method and experiences outlined in this paper offer a potential blueprint for educational institutions facing with similar challenges. 

## Ethics and consent

This study adheres to the ethical principles outlined in the **Declaration of Helsinki** and complies with the relevant institutional and national ethical guidelines governing educational research.

## Ethical approval

Since we analyze assessments structure and content in a learning management system, this study did not require formal ethical approval by the Ethical Review Board our local institution (Amsterdam University Medical Centre) or the Ethical Review Board (ERB) of the Dutch Association for Medical Education (Nederlandse Vereniging van Medisch Onderwijs, NVMO). According to the Amsterdam UMC institutional guidelines, research must be reviewed by the Medical Ethical Review Board (METC) if it qualifies as medical-scientific research under the Medical Research Act (Wet Medisch-wetenschappelijk onderzoek, WMO). Research is considered subject to WMO if there is any risk of physical or psychological harm to participants. In this study there are no participants, therefore does not have to be reviewed by the ERB of the Amsterdam UMC. If a study does not fall under the WMO but focusses on education, ethical approval by the NVMO ERB could be required.

The NVMO ERB policy states that ethical approval is not required as this study only involved collecting data on the structure and content of educational assessments from a learning management system, with no potential physical or psychological impact to individuals, This assessment was based on the following criteria:

- The data collected is on the structure and content of the assessments and learning goals.
- The data were collected as part of an existing quality assurance procedure within regular educational activities.
- No additional effort was required from lecturers beyond their routine duties.
- No personal or identifiable data were collected.
- There was no risk of harm or disadvantage to any individuals involved.

## Consent and participant considerations

This study did not involve human participants. The lecturers were not research participants but rather were engaged in their professional role as part of the routine quality assurance process. They were tasked by educational management to assess the impact of potential Generative AI (GenAI) use on learning goals and assessments. This was a standard aspect of their work responsibilities and would have occurred regardless of the study.

As no individual lecturer data were collected and no intervention was conducted, informed consent was not required. The model informed consent form from the NVMO requires to ask for consent if personal data is collected from the participants. The study did not involve surveys, interviews, or any additional data collection beyond existing curriculum materials (e.g., assignment descriptions from Act-e). All data used in this study were fully anonymized and could not be traced back to individuals.

Given that the research was conducted within the framework of routine educational quality assurance and complied with institutional and national guidelines, this study was exempt from formal ethical review.

## Confidentiality and data protection

All data were handled in accordance with the General Data Protection Regulation (GDPR) and the Amsterdam UMC data protection policies. Data storage complied with institutional regulations, ensuring that no individuals could be identified in any published results.

## Figures and Tables

**Table:** 

GenAI tool	Proprietary or open source	Software available from:
ChatGPT	Proprietary (with free options)	https://chatgpt.com/
Claude	Proprietary (with free options)	https://claude.ai/
Gemini	Proprietary (with free options)	https://gemini.google.com/
Mistral	Open Source Initative (OSI) approved open license software is under Apache 2.0-license.	https://mistral.ai/
Llama	Restricted Open License: Meta Llama 3.2 Community License	https://www.llama.com/

## Data Availability

Open Science Framework: Is your curriculum GenAI-proof? A method for GenAI impact assessment and a case study. 10.17605/OSF.IO/2YR6A. ( Jongkind *et al.*, 2025) The project contains the following underlying data: **MI2024 data AI impact scan** (GraphPad Prism file, in which the graphs were created). **Medical Informatics 2024 – results GenAI impact scan** (Excel file containing the same results as in the GraphPad Prism file to allow for more universal access to the data). The data is available under the terms of the Creative Commons Zero "No rights reserved" data waiver (CC0 1.0 Public domain dedication) ( http://creativecommons.org/publicdomain/zero/1.0/).

## Software availability

In the article the below generative AI tools are discussed. These tools can be accessed via the provided links. Archived versions of the software are not available.
