# Is your curriculum GenAI-proof? A method for GenAI impact assessment and a case study

**DOI:** 10.12688/mep.20815.2

**Published:** 2026-02-26

**Authors:** Remco Jongkind, Erik Elings, Erik Joukes, Tom Broens, Hemmik Leopold, Floris Wiesman, Jennita Meinema

**Affiliations:** 1 Teaching and Learning Centre, Amsterdam UMC Location AMC, Amsterdam, North Holland, 1105AZ, The Netherlands; 2 Medical Informatics, Amsterdam UMC Location AMC, Amsterdam, North Holland, 1105AZ, The Netherlands

**Keywords:** Generative AI, GenAI, Large Language Models, Curriculum adaptation, ChatGPT, assessment validity, AI literacy, learning outcome relevance.

## Abstract

**Background:**

Generative AI (GenAI) can complete tasks that previously could only be performed successfully by humans. Although GenAI provides many opportunities, it also poses risks such as invalid assessments and irrelevant learning outcomes. This article presents a broadly applicable method to (1) determine current assessment validity, (2) assess which learning outcomes are impacted by student GenAI use and (3) decide whether to alter assessment formats and/or learning outcomes. This is exemplified by a case-study on our Medical Informatics curriculum. We developed a five-step method to evaluate and address the impact of GenAI. Together with the respective lecturers, all assessments in the courses comprising a curriculum are analysed and subsequently adapted to address the impact of GenAI usage.

**Results:**

57% (55/96) of assessments, especially for courses involving writing and programming, were at risk of reduced validity and relevance. GenAI impact on assessment validity was closer related to the content and structure of assessments than to their complexity according to Bloom’s taxonomy. During educational retreats, lecturers discussed the relevance of impacted learning outcomes and whether students should be able to achieve them with or without GenAI. Furthermore, the results led to a plan to increase GenAI literacy and the intentional use by students during their education. For this purpose, course coordinators were asked to either adjust their manner of assessment to preclude GenAI use, or to alter learning outcomes to include GenAI use and literacy. For 64% of the impacted assignments (35/55) the assessment format was adapted and for 36% (20/55) the learning outcomes were adapted.

**Conclusion:**

The majority of assessments in our curriculum were at risk of reduced assessment validity and relevance of learning outcomes, necessitating adaptations of either the manner of the assessment or learning outcomes. The proposed method and case-study offer a potential blueprint for educational institutions facing similar challenges.

**List of abbreviations**
GenAI: Generative Artificial Intelligence
LLM: Large Language Models
MI: Medical Informatics
UvA: University of Amsterdam
NVMO: Nederlandse Vereniging voor Medisch Onderwijs - Dutch Society for Medical Education
GPT: Generative Pre-trained Transformer (specific to ChatGPT)

## Introduction

In recent years, the application of artificial intelligence (AI) has shown great potential to perform certain tasks faster or better than human professionals. Initially, AI was mainly used for the analysis of data, sound or image, therefore limiting its impact to certain professions and tasks. However, with the introduction of ChatGPT in November 2022, it became evident that generative artificial intelligence (GenAI) was rapidly evolving to also assist with or take over professions previously thought to be exclusively human, such as medical informaticians or doctors. GenAI, such as large language models (LLM), refers to AI systems that use input data, or prompts, to produce new content. This digital content most prominently includes text, images, and audio, although various other forms of content can be requested. The current article focuses primarily on LLMs, particularly the most used variant ChatGPT; a GenAI designed to generate coherent and contextually relevant text based on user prompts.

There are numerous examples showing that GenAI can take over and aid human processes in the workplace. For instance, Ayers *et al.* (2023) reported that ChatGPT v3.5 answered patients’ questions in an online forum more empathetically and accurately than doctors. ChatGPT v4, an upgraded version of ChatGPT v3.5, can effectively design, write and debug software code ( Azaiz *et al.*, 2023; Ellis *et al.*, 2024; Górecki, 2024; Idrisov & Schlippe, 2024; Kabir *et al.*, 2024; Kwon *et al.*, 2023; Palkowski & Gruzewski, 2024; Popovici, 2023; Reason *et al.*, 2024; Rodriguez *et al.*, 2024; Song *et al.*, 2024; Su & McMillan, 2024; Yeo *et al.*, 2024). ChatGPT can also be applied for modelling in health economics ( Reason *et al.*, 2024) and for reviewing mobile health apps ( Giunti & Doherty, 2024).

In the domain of university education, GenAI is poised to transform teaching and assessment practices, raising new questions about its potential and challenges. ChatGPT and other GenAI can be employed as a tool for many aspects of academic writing, such as for abstracts ( Cheng *et al.*, 2023; Hwang *et al.*, 2024), introductions ( Sikander *et al.*, 2023) medical reports ( Zhou, 2023), case reports ( Buholayka *et al.*, 2023), scientific reviews ( Kacena *et al.*, 2024; Nazzal *et al.*, 2024), cover letters ( Deveci *et al.*, 2023), patient educational material ( Mondal *et al.*, 2023), essays ( Herbold *et al.*, 2023), research articles ( Abuyaman, 2023; Huespe *et al.*, 2023; Lund & Ting, 2023; Saito & Tsukiyama, 2024; Semrl *et al.*, 2023) and peer reviews ( Saad *et al.*, 2024). Its performance in finding and using references, however, remains subpar as of now ( Lechien *et al.*, 2024; Mugaanyi *et al.*, 2024). Nonetheless, considering the rapidly evolving capabilities of GenAI, this feature might be improved in the near future, further qualifying it as a writing tool ( Dell’Acqua *et al.*, 2023).

GenAI has demonstrated the ability to pass various types of exams across educational domains. For example it has passed graduate level biomedical, physiology and biochemistry exams ( Ghosh & Bir, 2023; Stribling *et al.*, 2024; Subramani *et al.*, 2023), clinical informatics ( Kumah-Crystal *et al.*, 2023), graduate Python programming ( Ellis *et al.*, 2024), and a wide range of entry and specialist medical exams in a variety of countries such as Germany, the United States and the United Kingdom ( Cuthbert & Simpson, 2023; Farhat *et al.*, 2024; Griewing *et al.*, 2023; Kufel *et al.*, 2024; Li *et al.*, 2024; Lum, 2023; Meyer *et al.*, 2024; Sarangi *et al.*, 2024; Stengel *et al.*, 2024).

Previous examples show that GenAI has abilities that overlap with learning outcomes present in many curricula. Most particularly, skills in knowledge reproduction, scientific writing, programming, data analysis and communication seem to be impacted. This is also the case for our Medical Informatics bachelor curriculum, in which students learn to understand basic medical subjects, methodologies of medical-scientific research, software development and healthcare organization. This overlap allows students to use GenAI in their studies, as has also been reported at other universities ( De Winter *et al.*, 2023).

An informal anonymous poll among ~35 Medical Informatics students revealed that 95% admitted using ChatGPT in some form for their assessments. Another informal poll among ~100 of our third-year medical students provided a similar percentage (90%). This could be explained by students tending to choose the path of least resistance ( Abbas *et al.*, 2024). Uninformed use of GenAI is risky since students might be unaware of potential biases and incorrect answers that GenAI can sometimes provide. Furthermore, by using GenAI for assessments students might not achieve the intended learning outcomes relevant for their discipline.

Despite its potential, universities remain cautious about integrating GenAI into their curricula, citing concerns over academic integrity, assessment validity, and privacy. Additionally, the need to establish GenAI infrastructures that are accessible free of cost remains a significant challenge. Nevertheless, numerous universities have gradually started exploring pilot programs for GenAI management and usage. For instance, the University of Amsterdam (UvA) recently released policy guidelines stating that students may use GenAI if explicitly permitted for the specific course. Similarly, other universities have introduced policies stressing students’ responsibility for ensuring transparency and accountability in their work.

In mid-2023, some of the lecturers began to adjust their assessments and teaching methods to incorporate the new developments in GenAI. This, however, posed the risk of disrupting curriculum cohesion, as not all courses made an effort to adopt GenAI usage. For example, students might initially pass an assessment using GenAI in one course, only to face restrictions on its use in a later course, creating discrepancies in the skills needed for them to complete the assessment.

Therefore, we recognized the importance of evaluating the impact of GenAI on assessments and learning outcomes across entire study curricula. Our ultimate goal was to make any necessary adjustments to educational practices to ensure that assessments remain valid, learning outcomes stay relevant to future professional demands, and a consistent approach to GenAI use and policy is implemented. Ultimately, our goal is to safeguard the quality of the student learning experiences.

Before the start of this study (May 2023), only initial reporting on the impact of GenAI on the assessment or course level was available ( Chaudhry *et al.*, 2023). However, a comprehensive and consistent approach to the evaluation of its impact across an entire curriculum was lacking. At the time, the only curriculum-wide initiative present was a methodology being developed by van Ooijen-van der Linden *et al.* (2023), created to evaluate the impact of all forms of AI, not just GenAI. However, this methodology was still at an early stage of development, rendering it not yet usable for broader implementation. Additionally, it relied on time-consuming series of group discussions among lecturers, staff, and students, which presumably would not garner sufficient support from our teaching faculty. Informal inquiries within the Dutch Society for Medical Education (NVMO) working group on academic development and the Teaching and Learning Centres at other UvA faculties also failed to yield suitable methodologies. Consequently, we decided to develop our own method to assess the curriculum wide impact of GenAI on assessment validity and learning outcome relevance.

In March 2023, it was hypothesized that assessments involving writing of non-academic products outside the classroom would be most impacted, followed by academic products and software coding. We also expected that due to its hallucinations (i.e., false information presented as facts), GenAI would perform worse at the “lower” Bloom levels of remembering and understanding since this is fact-based. Most accurate performance was anticipated for the intermediate Bloom levels of applying and analysing, deteriorating again at the higher levels of creating and evaluating. Therefore, it was expected that GenAI would be able to complete more assessments in year 1 compared to year 3 due to the increase in Bloom levels of learning outcomes over the years in our curriculum.

This article outlines the method and the results of the GenAI impact scan conducted on the Medical Informatics bachelor curriculum as a case study. It also details the steps we have taken to proof the curriculum for future developments, along with practical recommendations for other educational programs based on our experiences.

### GenAI impact scan method – determining the impact on assessment validity and relevance of learning outcomes

In March 2023, five months after the launch of ChatGPT3.5, a five-step method was developed and subsequently implemented to evaluate the impact of GenAI at a curriculum level. The curriculum was adapted accordingly to preserve the constructive alignment of the courses. This method aimed to identify which types of assessments and learning objectives are most impacted by student use of GenAI.

To address this issue, a multi-step approach was established ( Table 1). First, the urgency for action was determined (Step 1) to estimate the extent of the impact and create awareness and willingness for change among staff. Next, assessments where GenAI use was deemed impossible, such as on-site and practical examinations, were excluded (Step 2). This process incrementally funnels the learning assessments on their risk of being invalidated by GenAI and reduces the number of assessments up for evaluation, and hence the workload in later steps. Afterward, lecturers were consulted as experts on the subject matter to evaluate potential impact on their course (Step 3). To ensure coherent curriculum adjustments, a collaborative discussion with the lecturers was conducted (Step 4). Finally, given the rapid evolution of GenAI capabilities ( Dell’Acqua *et al.*, 2023), Steps 2, 3 and 4 are planned to repeat annually for each learning assessment.

**Table 1.** Table describing the five steps of the GenAI impact scan method with the goal, the input needed for the step, the outcomes of the step and the involved actors. GenAI: Generative AI, PDCA: Plan-Do-Check-Act.

	Goal	Input	Outcomes	Actors
** Step 1**	Estimating the extent to which learning activities are impacted to determine urgency	Set of learning activities used in the curriculum, such as workplace learning, projects, essays, etc.	Set of used learning activities that are impacted by GenAI	Educational advisors
**Step 2**	Determining whether students have the opportunity to make the assessment with GenAI. This reduces the number of assessments that lecturers need to screen.	Set of assessments in the curriculum	Score that expresses the possibility of GenAI abuse per assessment. (No risk/Partial risk /High risk).	Educational advisors
**Step 3**	Determining to which degree GenAI can complete the assessments. This is to decide which assessments or learning outcomes to alter.	Set of (partially) at risk assessments in the curriculum	Score that expresses the degree of ability of GenAI to successfully complete the assessment per assessment (partially) at risk.	Lecturers, educational advisors
**Step 4**	Determining if and how to adapt the assessments or learning outcomes	Set of assessments risk scores per assessment, set of affected learning outcomes of all courses. Exit competences were not considered due to their general nature.	Decision per learning goal whether to keep, adapt or remove it. List of new learning outcomes that we wish to add to the curriculum, for example AI literacy.	Lecturers, educational advisors, educational management
**Step 5**	Ensuring the ongoing validity and relevance of assessments and learning outcomes by repeating step 2 and 3 yearly in the PDCA cycle.	The assessments per course	For each assessment an indication whether it is at risk and a score that expresses the degree of ability of GenAI to successfully complete the assessment.	Lecturers

### Step 1: Pre-scan

In the first step, common educational formats are evaluated at a curriculum level and estimations are made on the degree to which GenAI can be used by students to complete these assessments. This step functions to give an initial impression of the impact of GenAI and to determine the urgency of the GenAI impact scan. Examples of such educational formats include team-based learning, project-based education, and workplace learning.

We expect project- and case-based reports, programming take-home exams and academic writing to be aspects most impacted, since these assessments often occur without direct supervision and involve actions that GenAI is proficient in.

### Step 2: Quick scan

For the second step, educational advisors identify assessments at risk of students using GenAI undetected to complete summative tasks, thereby failing to achieve the intended learning outcomes. Educational advisors evaluate all summative assessments within the curriculum, answering the following 3 questions for each assessment:

• 1. Is the assessment conducted under supervision?
When an assessment takes place under supervision, the likelihood of students displaying academic dishonesty is much lower ( Janke *et al.*, 2021; Yazici *et al.*, 2011).
• 2. Does the course require intermediate products to be submitted before the final assessment?
Intermediate products have been employed by many leading universities such as Caltech University, Cornell University and University of Tokyo to improve the authenticity and validity of the eventual summative assessments ( Moorhouse *et al.*, 2023).
• 3. Is the weight of the assessment <10% of the final grade of the course?
This question was mainly asked to prioritize which assessments to analyse and if necessary, to adapt first.

If the answers to all three above questions are no, then the assessment is classified as *high-risk.* If the answer to question 1 is no, then the assessment is classified as *partial risk.* Otherwise, the assessment is classified as *no risk.* For all high or partial risk assessments a detailed scan is performed with priority being given to the high-risk assessments.

### Step 3: Detailed scan

In the third step lecturers responsible for the assessments conclude whether the assessments that are deemed at risk can in fact be made with GenAI.

In this step, two hands-on workshops are organized for the course coordinators and lecturers in which basic knowledge of GenAI and effective prompt writing is taught. For this workshop, the coordinators and lecturers should be provided access to a GenAI tool, which ideally mirrors one that students are likely to use. Additionally, it should preferably use an advanced frontier large language model, should not train on input data and should have functionalities such as uploading files, creating images, using plugins, and creating custom chatbots such as GPTs in ChatGPT.

In these workshops, lecturers attempt to complete their own at-risk learning assessments with the help of GenAI. If they do not manage to complete all these assessments during the workshops they are asked to continue later and report to educational advisors via a central form within a predefined period of time.

The lecturers are asked to classify their assessments on a Likert scale of 1 to 5 with levels as presented below. These labels were created by the educational advisors and tested for suitability on one first-year bachelor’s course and one first-year master’s course in medical informatics. These courses were chosen as representative for the curriculum by the educational management. Based on the initial tests, minor adjustments were made to the levels.

Rating scale:

1. GenAI provides a perfect or very good output that can be used for the assessment. The prompt needed for GenAI to produce this output (almost) literally matches the assessment description.
2. GenAI provides a perfect or very good output that can be used for the assessment. The prompt needed for GenAI to produce this output has to be refined based on the assessment description. This requires knowledge of how to prompt effectively.
3. GenAI provides an insufficient solution based on solely the assessment description and effective prompts. Additional subject matter knowledge or skills are necessary for a perfect or good assessment product.
4. GenAI provides an insufficient output for the assessment, even with effective prompting and additional subject matter knowledge or skills. GenAI can only be used as inspiration; for example to brainstorm.
5. It is not possible to use GenAI for this assessment; for example, because actions must be taken in certain software systems which GenAI cannot access.

The rating scale provides a measure of resistance a student may experience when using GenAI to help with an assessment, as students tend to choose the path of least resistance ( Abbas *et al.*, 2024). For example, for an assessment where (almost) literal copying of the assessment description results in a good outcome (level 1), the threshold for using GenAI is lower compared to an assessment that requires modifications to the prompt to achieve a good result (level 2). Therefore, we expect that each level on the scale represents a (non-linear) increase in usability and probability for a student to utilize GenAI for the assessment. Furthermore, the score indicates which skills or knowledge the students still need to complete the assessment, namely GenAI literacy skills or subject matter expertise.

After rating all learning assessments in the curriculum on their level of AI usability, adjustments can be prioritized. The educational management decided that scores 1, 2 and 3 for assessments were undesirable and that either the manner of assessment or the learning outcomes should be adapted.

### Step 4: Curriculum adaptation

Assessments with a score of 1, 2, and 3 in step 3 and their associated learning outcomes are categorized in clusters based on type of skill assessed.

Cluster examples include:

1. Academic skills
2. Programming
3. Writing skills (academic and non-academic)
4. Meta-cognitive skills
5. Data (synthesizing, analysis, etc.)

Subsequently, the impact of GenAI on the learning outcomes is discussed with all lecturers. The lecturers are divided into groups according to their expertise and asked to determine for each of their assigned learning outcomes whether they would like to start, adapt or continue teaching certain learning outcomes considering the advances in GenAI. In this context, “start” implies the addition of new learning goals to the curriculum (e.g. AI literacy), “adapt” implies adding GenAI use to the learning outcome and “continue”implies continuing to teach the skill without any adaptation for GenAI advances. For the category “adapt” the lecturers should indicate from which year of the study GenAI use should be allowed for that specific learning outcome. This ensures that the students learn basic knowledge and skill without GenAI, enabling them to critically evaluate the output when later allowed to use GenAI. This method is an adjusted format of the Start, Stop, Continue methodology from Cowan & George (1999).

Based on the output gathered from this meeting, the educational management formulates a policy for the coherent adjustment and addition of learning outcomes to the curriculum.

Subsequently, course coordinators can then refer to this policy to adapt their at-risk assessments and learning outcomes. These adaptations fall into one of two categories:

• Change the assessment format to prevent GenAI usage, ensuring students achieve the learning outcomes independently.
• Adjust the learning outcomes to better reflect the (desired or future) situation in the workplace. This requires the learning outcomes, assessment and teaching to be adapted so that the students can achieve the new or adapted learning outcomes: for example, by learning how to make a business plan for an e-health app in co-creation with GenAI.

### Step 5: Consolidation

Since the abilities of GenAI are rapidly improving ( Dell’Acqua *et al.*, 2023), it is important to keep evaluating the impact of GenAI use by students on assessment validity and learning outcome relevance.

Therefore, the quick scan (step 2) and the detailed scan (step 3) are included in the Plan Do Check Act (PDCA) cycle of each course.

**Figure 1.**
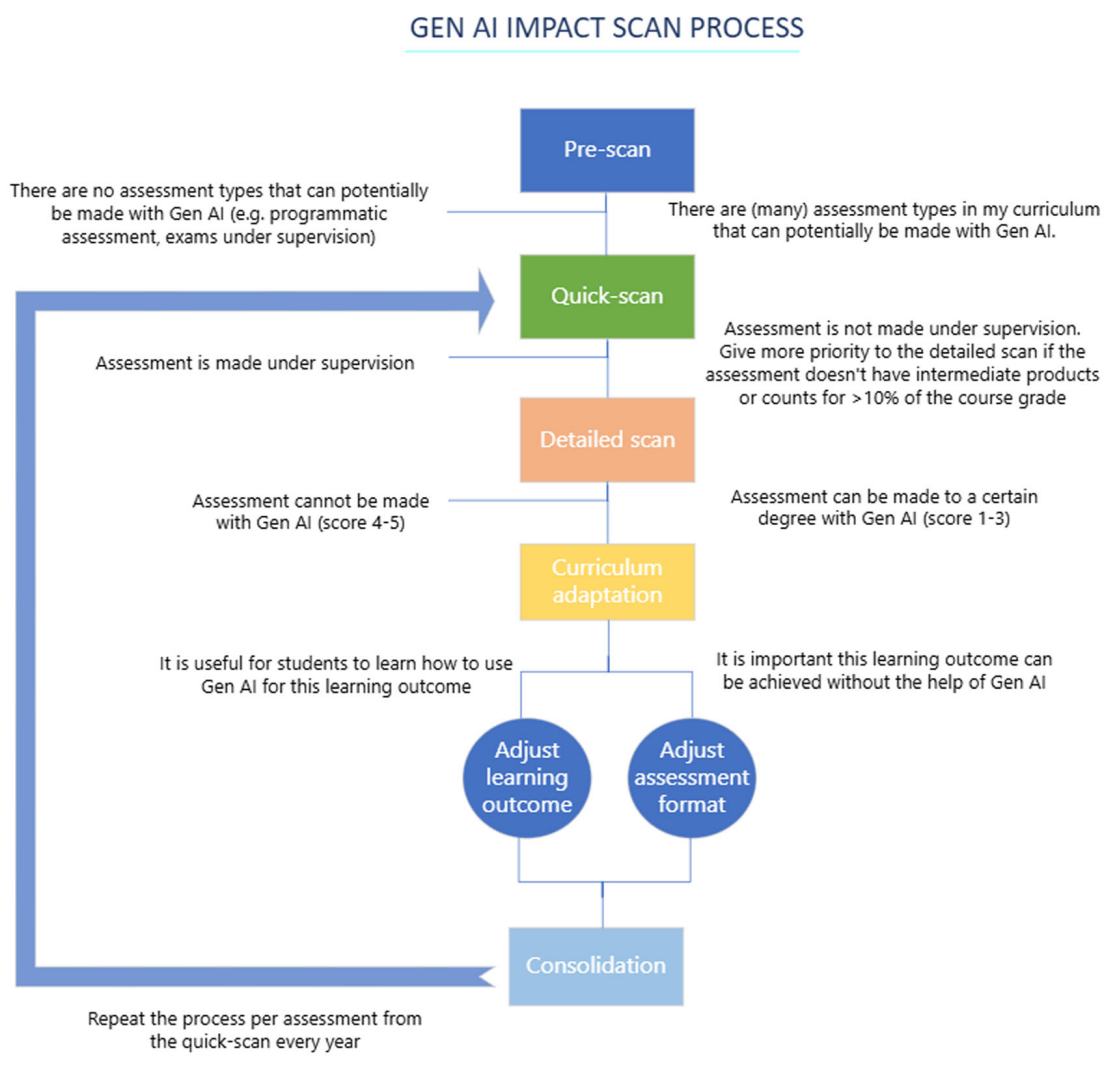
Process flow chart, presenting the questions to be answered or decisions to be made for each step.

The process and decisions to be made during the five steps as described above are visualized in the process flow chart in Figure 1.

## Results – a case study from the Bachelor of Medical Informatics

**Figure 2.**
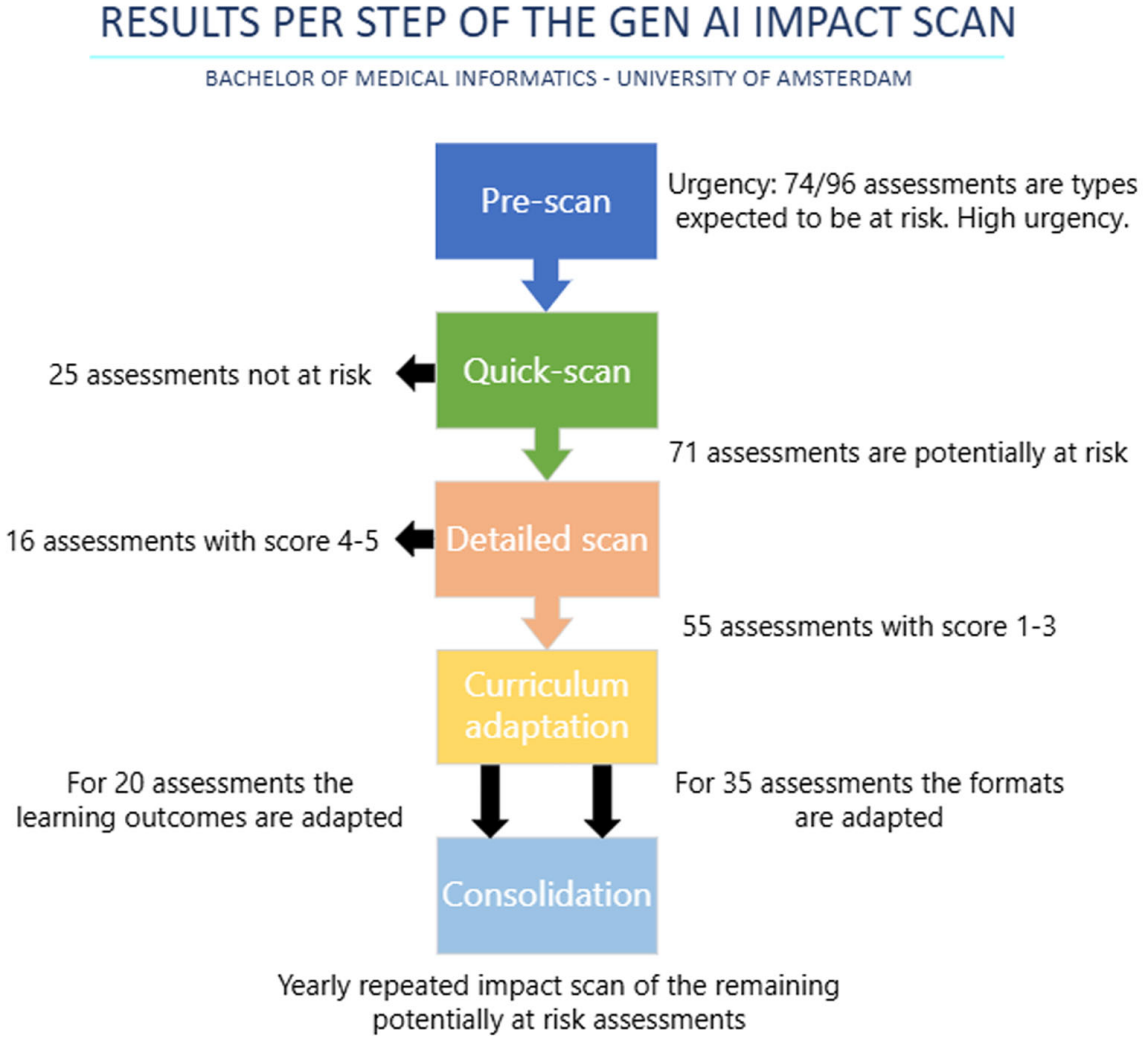
Flowchart describing the five steps of the impact scan method with the results per step.

In the following section the case study from our own Medical Informatics bachelor curriculum will be highlighted to give an indication of the possible impact of student GenAI usage on the validity of assessments and relevance of learning outcomes. The results from this case study are summarized in Figure 2. The curriculum consists of a total of 16 courses, 169 learning outcomes and 96 assessments.

### Step 1 Pre-scan

With the pre-scan, the characteristic elements of the Medical Informatics curriculum that would likely be impacted by GenAI were identified. These elements were gathered from our curriculum database Act-e (Broens *et al.*, 2017). This is a database outlining all learning goals, learning activities and assessments in the curriculum.

Subsequently, it was estimated to which degree these activities, or their assessments, are impacted by GenAI based on our personal experiences with the capabilities of GenAI and earlier literature ( Dell’Acqua *et al.*, 2023; George *et al.*, 2023; Kalla *et al.*, 2023). Workplace learning, for example, such as in medical residency, is relatively unaffected by GenAI as it primarily involves hands-on activities and verbal interactions, with minimal reliance on written assessments by the student.

Our bachelor uses 19 different assessment types. Given that 74 out of 96 assessments (77%) in the bachelor’s program were project reports, academic writing, or programming take-home exams—types considered more vulnerable to GenAI—the next steps of the AI impact scan were considered more urgent.

### Step 2 Quick scan

After identifying the learning activities that may be impacted by GenAI during the pre-scan, it became a high priority to determine which specific assessments were at risk of being completed with GenAI. This led to the implementation of the quick scan, where to focus was on identifying assessments vulnerable to unnoticed use of GenAI by students.

Educational advisors determined which assessments were at risk because students had the opportunity to complete summative assessments unnoticed using GenAI and therefore potentially not reaching the required learning outcomes.

In sum, a total of 71 (74%) assessments were deemed (partially) at risk for our bachelor curriculum. This included 57/96 (59%) summative assessments at partial risk and 14/96 (15%) which were deemed high-risk.

These high numbers aligned with expectations based on the pre-scan because of the high amount of project and case-based education in the curriculum. Most notably, the academic reports, business plans, advice reports and take-home exams for programming were scored at risk.

### Step 3 Detailed scan

Having established which assessments were potentially at risk through the quick scan, a more in-depth analysis was necessary to understand the extent of this risk. In the detailed scan, lecturers were consulted to evaluate how and to what degree GenAI could be used to complete these learning assessments.

For this purpose, lecturers were offered 2 workshops during which they were trained in the use of GenAI. Subsequently, lecturers were provided with ChatGPT Team licences, which were GDPR-compliant and no training was done on the input data. During and following the workshop the lecturers determined on the 1-5 scale which of the (partially) at risk learning assessments could in fact be completed with GenAI. We selected ChatGPT (version 4) over other GenAI tools such as Claude, Gemini, Mistral, and Llama, assuming most students would use it due to its strong brand recognition. Additionally, ChatGPT consistently ranked as the most capable tool on LLM benchmark boards, such as UC Berkeley’s LMSys Chatbot Arena, during the study period (April 2023–June 2024).

The five-point rating criteria as discussed in the method section were applied. Fifty-five of the 71 at-risk assessments had a score between 1 and 3 and could therefore to a certain degree be completed by GenAI. Thus, 55 out of the total 96 assessments (57%) in our curriculum were at risk of reduced assessment validity or learning goal relevance.

**Figure 3.**
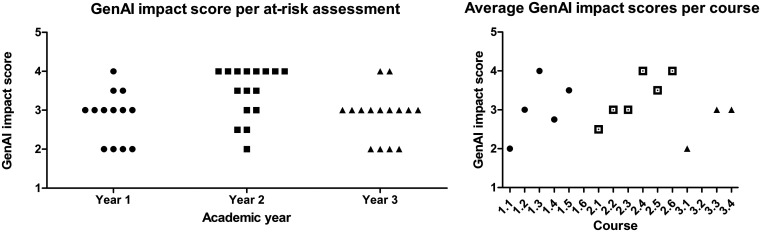
Two graphs depicting the GenAI impact scores assigned by lecturers, indicating to which degree GenAI can be used to make the assessments, per year (left) or course (right). With 1 being GenAI able to completely make the assessment and 5 that no GenAI usage is possible. For a detailed description of the interpretation of each score see step 3 in the method section. The courses where no impact score is indicated had no assessments where students have the opportunity to make the assessments with GenAI. Each dot in the left graph represents an assessment, each dot in the right graph represents an average of the assessments across a course. An assessment can have a non-integer score if some elements of the assessment fall in one score bracket and some in the other.

Over the entire curriculum, an average AI impact score of 3.1/5 was found. This varied between year 1 (2.8/5), year 2 (3.5/5) and year 3 (2.9/5) ( Figure 3, left). The average scores of at-risk assessments per course are displayed in Figure 3 on the right. Following the plenary meetings, the results of these assessments were discussed one-on-one by the curriculum committee with the coordinating staff of all courses in the curriculum.

### Step 4 Curriculum adaptation

With the detailed scan revealing which assessments could indeed be (partially) completed using GenAI, it became necessary to address this identified impact. In response, we undertook a process of adapting the curriculum to mitigate the impact of GenAI on learning outcomes, resulting in a series of policy changes, assessment adjustments and learning goal adaptations.

The first step in this process was to cluster the learning outcomes into the following six categories:

1. Subject matter knowledge
2. Academic skills
3. Programming
4. Writing skills (academic and non-academic)
5. Meta-cognitive skills
6. Data (e.g., synthesizing, analysis)

Subsequently, an educational afternoon retreat of 3 hours with all the medical informatics lecturers was organized. Groups were assembled according to field of expertise and asked to determine for each of their assigned learning outcomes whether they would like to start, adapt or continue teaching these learning outcomes to students considering the advances in GenAI. Reasons mentioned to allow GenAI use for learning goals (“adapt”) were preparing students for the future workplace and teaching students AI literacy. Reasons for not allowing students to use GenAI for learning goals (“continue”) were the inherent value of a process, for example writing to learn to structure thoughts, and acquiring sufficient knowledge and understanding of the material to be able to critically evaluate the output. Each group was chaired by an educational advisor or a programme director.

Based on the input from this afternoon retreat, the curriculum committee defined a policy on the usage of GenAI by students throughout the Bachelor of Medical Informatics.

The majority of the learning outcomes (~80%) were indicated by lecturers as important to continue teaching, as these covered basic knowledge and were necessary to assess the correctness of the output of GenAI. For the remaining ~20% of the learning outcomes, lecturers indicated that these would benefit from (partial) adaptations to include GenAI use. Several learning outcomes were added, mainly pertaining to GenAI literacy and skills. These learning outcomes should enable students to effectively and responsibly use GenAI regarding privacy, data security, environmental impact, academic integrity, biases, didactic considerations (e.g., mental complacency) and output accuracy.

In line with the policy of the University of Amsterdam, educational management recommends that first-year coordinators should limit or avoid the use of GenAI to ensure students establish a solid foundation in medical informatics. During this time, students are also trained to critically evaluate GenAI output for reliability and accuracy. In the second and third years, coordinators are encouraged to explore AI use for the learning goals and its potential to enhance learning, while ensuring it does not fully replace traditional methods. Furthermore, it is important that lecturers including the use of GenAI for the development of a specific skill clearly communicate this use to later lecturers in order to maintain constructive alignment across the curriculum. As a general principle, students should be informed that the use of GenAI is not permitted unless its permission for use in assessments is explicitly stated in the course manual.

The most common changes to learning assessments are regarding the assessment format, precluding the use of GenAI. Out of the 55 assessments with an assigned impact score of 1–3, for 35 assessments (64%) the format was changed by the course coordinators. These changes most often included examinations under supervision, changing a paper to a presentation or podcast, making the assessment process-focused and adding a number of (formatively) assessed intermediate products.

For the remaining 20 out of 55 assessments (36%) with a score of 1–3, which mainly included assessments in programming and non-academic writing, learning outcomes were altered by the course coordinators.

The most common adjustments to learning outcomes included adjusting assessment weightings in grading rubrics, incorporating AI literacy, raising the difficulty of learning outcomes, and rephrasing outcomes to integrate GenAI usage.

### Step 5 Consolidation

Once the curriculum had been adapted to the GenAI impact, a process for continuous adaptation was required as GenAI capabilities are rapidly increasing ( Dell’Acqua *et al.*, 2023). This was established in the consolidation step, during which a long-term strategy was developed to guarantee that the curriculum remains aligned with evolving GenAI capabilities.

To ensure continuous adjustment, the quick scan (step 2), detailed scan (step 3) and curriculum adaptation (step 4) are integrated into the Plan Do Check Act (PDCA) cycle for each course. In a yearly exam evaluation report, course coordinators are asked questions on whether students can complete their assessments using GenAI and if so, what the proposed changes are.

The answers to these questions will then be discussed in a subsequent “action plan” meeting between the course coordinator and the educational management preceding the start of each course.

As this last analysis will be implemented in the upcoming academic year, no data is available to illustrate this step as of now.

## Conclusion & discussion

The growing use of GenAI by students has necessitated a revaluation and adjustment of assessments and learning outcomes of curricula. This article describes a generalizable method to determine the validity of assessments and impact of GenAI on learning outcomes. It also proposes a process to systematically decide whether to alter the assessment formats or the learning outcomes. This approach is illustrated through a case study on the medical informatics curriculum revealing that 57% (55/96) of the learning assessments could be completed to a certain degree using ChatGPT.

**Figure 4.**
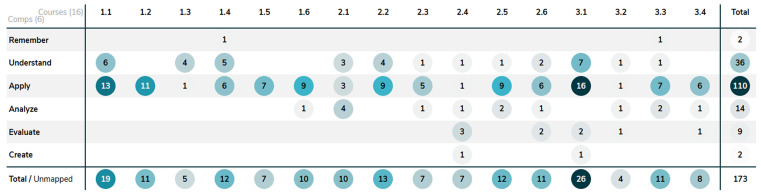
Shows the distribution of the complexity of learning outcomes, categorized by Bloom's taxonomy, across the curriculum database Act-e for courses 1.1–3.4. The first number represents the year of the course, and the second represents the order of the course within that year.

Contrary to our initial hypothesis, the usability of ChatGPT did not decrease in year 3 compared to year 1 of the bachelor whereas the Bloom levels of the assessments do increase ( Figure 4) as students progress. The average GenAI impact score was 2.9 in year 1, 3.5 in year 2, and 2.8 in year 3. Bloom levels do not appear to determine the performance of a specific learning assessment on our GenAI impact scale; conversely, the content or manner of assessment formulation seems to be of importance. Furthermore, the assessments were made with ChatGPT v4, which provides considerably fewer hallucinations than ChatGPT v3.5 and has higher performance in general (e.g. Chelli *et al.*, 2024), thus potentially improving its usability at lower Bloom levels. Similarly, as of spring 2023, other studies have been published showcasing either no difference in the performance of ChatGPT across different Bloom levels or an improved performance at lower Bloom levels ( Ghosh & Bir, 2023; Herrmann-Werner *et al.*, 2024).

The essential challenge within a curriculum is to decide which learning outcomes students should achieve without GenAI to ensure they acquire basic knowledge, and which learning outcomes should allow students to use GenAI. For this, it is important to discern various types of GenAI use which may be applicable to the different learning outcomes. For instance, more basal learning outcomes, such as the ability to produce grammatically accurate text in a scientific tone, can be achieved through GenAI without significant consequences to academic integrity. Such usage is comparable to more traditional manners of superficial text reviewing, like human copy editors or built-in spell checker extensions. As these functions primarily aid with the format, but not the content, the requirement for the student to demonstrate critical, original thinking remains. Employing GenAI tools to produce original content, conversely, poses a risk for adequate academic development and independent functioning in the future workplace. It is up to lecturers to decide to what extent this manner of usage is acceptable for their learning outcomes in step 4. The decision should also take into account the consistency of learning goals across the curriculum. Research shows that students with access to GenAI reach performance scores up to 48 to 127% higher than students without access to GenAI. However, when presented with a similar task at a later assessment without the ability to use GenAI, the students that used GenAI score 17% lower than students who did initially not have access to GenAI ( Bastani *et al.*, 2024). The higher performance of students using GenAI, illustrates that prohibiting the use of GenAI is not effective, since students can be expected to have to use GenAI in the workplace to be competitive ( Dell’Acqua *et al.*, 2023). It is however important that such GenAI access is consistent or increases incrementally over the course of a curriculum, to prevent the setback experienced by students who acquired a skill using GenAI but are no longer allowed to use it in following courses. When deciding to allow GenAI use for learning assessments, students need to be educated to effectively and responsibly use GenAI with regards to privacy, data security, environmental impact, academic integrity, biases, output accuracy and didactic considerations such as mental complacency.

There are some important considerations and notes regarding the case study. First, since our main aim was to adapt the curriculum and ensure adequate learning of skills and knowledge by our students, several learning assessments were already adjusted before or during our study. Consequently, the results provided in the case study do not represent a completely “pre-GenAI” curriculum and the reported impact of GenAI on the validity and relevance of assessments will be an underestimation.

Additionally, the process by which lecturers evaluated the capability of ChatGPT to complete their assessments is subjective and dependent on the lecturers’ GenAI proficiency. To mitigate this subjectiveness and skill dependency, we provided two workshops in which we trained the lecturers in GenAI use. We also encouraged the lecturers to collaborate during the workshops on the assessments and discuss their verdict with each other and the educational advisors. Since most lecturers are medical informaticians with high digital literacy, they were well-prepared to engage with GenAI. However, when applying this method to other curricula, more extensive GenAI training may be necessary. A potential improvement could involve having student assistants attempt the assessments with GenAI to better simulate students’ proficiency with both the subject matter and GenAI.

Beyond the impact on the learning outcomes and the (future) work field, GenAI also offers great opportunities and considerations for lecturers and students during the study itself.

For instance, several studies show that GenAI tools can assist students to develop certain skills, such as academic writing( Kurniati & Fithriani, 2022; Wang, 2020; Zhao, 2023). However, other studies demonstrate or predict negative effects such as the development of cognitive biases, lower grades due to assessors’ prejudices against the use of AI, the required extra attention for critical appraisal of sources, and cognitive complacency ( Abbas *et al.*, 2024; Choudhury & Shamszare, 2023; Liu *et al.*, 2022; Lund & Wang, 2023). Therefore, it is important that more research is done regarding the potential cognitive effects of GenAI use on learning, specifically on which guardrails would work best to provide optimal learning. GenAI applications that don’t instantly give away answers but provide the right amount of challenge to encourage students to think, interact with the material and allow for reflection could be a solution ( Bastani *et al.*, 2024).

To ensure students achieve the intended learning outcomes without GenAI a shift is needed from traditional written assessments to either supervised or more interactive forms of assessment, such as presentations, in-person exams, or process-focused projects that are difficult for AI to replicate ( Bower *et al.*, 2024; Chaudhry *et al.*, 2023; Kaldaras *et al.*, 2024; Zirar, 2023). A challenge this solution poses is that such methods generally tend to be more time consuming for faculty and more resource intensive, requiring examination or lecture rooms. Moreover, it should be acknowledged that writing assignments in their traditional way contribute significantly to academic development by providing a distinctive educational value apart from solely functioning as an assessment method. This is illustrated by a substantial multi-institutional study by Anderson *et al.* (2015), who found that effective academic writing practice gives shape to fundamental skills which allow for deeper learning and higher perceived gains in learning and development. Such skills include the evaluation of sources, synthesizing theories on a more complex level, perspective-taking and application of learned knowledge in other contexts, as well as more practical, personal and social skills of problem-solving and eloquence. Likewise, being able to critically revise written text is an important skill at risk when different assessment types are implemented. For instance, exam assessments might rely predominantly on memorization and often do not allow for critical revision and integrative discussion of topics across different perspectives. This removes the interactive and meaning-making aspect of effective writing practice, as proposed by the literature. Writing additionally motivates the student to demonstrate unique thinking skills, enhancing creativity and individuality ( Herrington, 1981).

For above-mentioned reasons, some writing assessments should be maintained in their original format, instead adapting learning goals to integrate GenAI usage. In this case, it is important to account for the potential increase in difficulty and scope of learning assessments, as students using GenAI could be expected to produce more extensive output. To prevent inequity, it is essential to provide students with equal access to GenAI platforms, ensuring that those unable to afford GenAI subscriptions are not disadvantaged.

In conclusion, our findings advocate for a systematic and adaptive approach to curriculum development in response to GenAI use and its impact on assessment validity and relevance. The method and experiences outlined in this paper offer a potential blueprint for educational institutions facing similar challenges.

## Ethics and consent

This study adheres to the ethical principles outlined in the **Declaration of Helsinki** and complies with the relevant institutional and national ethical guidelines governing educational research.

## Ethical approval

Since we analyze assessments structure and content in a learning management system, this study did not require formal ethical approval by the Ethical Review Board of our local institution (Amsterdam University Medical Centre) or the Ethical Review Board (ERB) of the Dutch Association for Medical Education (Nederlandse Vereniging van Medisch Onderwijs, NVMO). According to the Amsterdam UMC institutional guidelines, research must be reviewed by the Medical Ethical Review Board (METC) if it qualifies as medical-scientific research under the Medical Research Act (Wet Medisch-wetenschappelijk onderzoek, WMO). Research is considered subject to WMO if there is any risk of physical or psychological harm to participants. In this study there are no participants, therefore does not have to be reviewed by the ERB of the Amsterdam UMC. If a study does not fall under the WMO but focusses on education, ethical approval by the NVMO ERB could be required.

The NVMO ERB policy states that ethical approval is not required as this study only involved collecting data on the structure and content of educational assessments from a learning management system, with no potential physical or psychological impact to individuals. This assessment was based on the following criteria:

• The data collected is on the structure and content of the assessments and learning goals.
• The data were collected as part of an existing quality assurance procedure within regular educational activities.
• No additional effort was required from lecturers beyond their routine duties.
• No personal or identifiable data were collected.
• There was no risk of harm or disadvantage to any individuals involved.

## Consent and participant considerations

This study did not involve human participants. The lecturers were not research participants but rather were engaged in their professional role as part of the routine quality assurance process. They were tasked by educational management to assess the impact of potential Generative AI (GenAI) use on learning goals and assessments. This was a standard aspect of their work responsibilities and would have occurred regardless of the study.

As no individual lecturer data were collected and no intervention was conducted, informed consent was not required. The model informed consent form from the NVMO requires to ask for consent if personal data is collected from the participants. The study did not involve surveys, interviews, or any additional data collection beyond existing curriculum materials (e.g., assignment descriptions from Act-e). All data used in this study were fully anonymized and could not be traced back to individuals.

Given that the research was conducted within the framework of routine educational quality assurance and complied with institutional and national guidelines, this study was exempt from formal ethical review.

## Confidentiality and data protection

All data were handled in accordance with the General Data Protection Regulation (GDPR) and the Amsterdam UMC data protection policies. Data storage complied with institutional regulations, ensuring that no individuals could be identified in any published results.

## Figures and Tables

**Table:** 

GenAI tool	Proprietary or open source	Software available from:
ChatGPT	Proprietary (with free options)	https://chatgpt.com/
Claude	Proprietary (with free options)	https://claude.ai/
Gemini	Proprietary (with free options)	https://gemini.google.com/
Mistral	Open Source initiative (OSI) approved open license software is under Apache 2.0-license.	https://mistral.ai/
Llama	Restricted Open License: Meta Llama 3.2 Community License	https://www.llama.com/

## Data Availability

Open Science Framework: Is your curriculum GenAI-proof? A method for GenAI impact assessment and a case study. 10.17605/OSF.IO/2YR6A. ( Jongkind *et al.*, 2025) The project contains the following underlying data:
•
**MI2024 data AI impact scan** (GraphPad Prism file, in which the graphs were created). •
**Medical Informatics 2024 – results GenAI impact scan** (Excel file containing the same results as in the GraphPad Prism file to allow for more universal access to the data). The data is available under the terms of the Creative Commons Zero “No rights reserved” data waiver (CC0 1.0 Public domain dedication) ( http://creativecommons.org/publicdomain/zero/1.0/).

## Software availability

In the article the below generative AI tools are discussed. These tools can be accessed via the provided links. Archived versions of the software are not available.
