# Supplementary material for: Is your curriculum GenAI-proof? A method for GenAI impact assessment and a case study
Source: MedEdPublish (2016). 2026 Apr 28;15:11. Originally published 2025 Mar 26. [Version 3] doi: 10.12688/mep.20815.3 (PMC12976610; doi:10.12688/mep.20815.3)
Supplement: Peer review history

# Reviewer report: Reviewer response for version 3

**DOI:** 10.21956/mep.23136.r45800

**Published:** 2026-05-06

**Authors:** Suriyaarachchige Nishan Silva

**Affiliations:** 1 CHI Health, Claremont, Australia; 2 Fellowship Support Program, Royal Australian College of General Practitioners, East Melbourne, Victoria, Australia

Thank you for accepting and including the suggested amendments constructively. This has strengthened the robustness of the flow and the content. Particularly, the changes made at the introduction have created a more impactful opening to the paper.

Is the rationale for developing the new method (or application) clearly explained?

Partly

Is the description of the method technically sound?

No

Are the conclusions about the method and its performance adequately supported by the findings presented in the article?

Partly

If any results are presented, are all the source data underlying the results available to ensure full reproducibility?

Partly

Are sufficient details provided to allow replication of the method development and its use by others?

No

Reviewer Expertise:

Health Informatician, Health professionals educationist

I confirm that I have read this submission and believe that I have an appropriate level of expertise to confirm that it is of an acceptable scientific standard.

# Reviewer report: Reviewer response for version 3

**DOI:** 10.21956/mep.23136.r45801

**Published:** 2026-05-04

**Authors:** Nilesh Kumar Mitra

**Affiliations:** 1 IMU University, Kuala Lumpur, Malaysia

In this article, "A method for GenAI impact assessment and a case study", the authors have developed a five-step method to evaluate and address the impact of GenAI on assessment validity. The authors have involved several course coordinators to carry out the project and concluded that majority of assessments conducted using GenAI in their curriculum were at risk of reduced validity and this was related to the nature of learning outcomes. Following the last review the authors have added additional information on published literature under introduction to bring clarity to the research question. The authors have also described the 5th step consolidation in greater detail.

Is the rationale for developing the new method (or application) clearly explained?

Partly

Is the description of the method technically sound?

Yes

Are the conclusions about the method and its performance adequately supported by the findings presented in the article?

Yes

If any results are presented, are all the source data underlying the results available to ensure full reproducibility?

Yes

Are sufficient details provided to allow replication of the method development and its use by others?

Partly

Reviewer Expertise:

Technology enhanced learning, AI, Small group Learning, Online assessment

I confirm that I have read this submission and believe that I have an appropriate level of expertise to confirm that it is of an acceptable scientific standard.

# Reviewer report: Reviewer response for version 2

**DOI:** 10.21956/mep.23018.r45469

**Published:** 2026-03-10

**Authors:** Nilesh Kumar Mitra

**Affiliations:** 1 IMU University, Kuala Lumpur, Malaysia

The article "Is your curriculum GenAI-proof? A method for GenAI impact assessment and a case study"  has been submitted under new educational method. The authors have claimed that they have developed a five step method to evaluate and address the impact of Generative AI on assessment. From the literature review described in the article, it is evident that comparable literature of impact assessment is not significant in number. 

 It is clear that the authors could not append enough evidence about GenAI's impact on assessment and learning outcomes. The academic literature should describe the necessity of a research or study based on evidence available in the published literature.

 The authors hypthothesized that GenAi would perform worse at the lower Bloom's taxonomy of remembering and understanding. There is no citation available following this hypothesis. Academics should not append assumptions in the scientific literature. In 2023 Australian Journal of Educational Technology published an article written by Nguyen Thanh et al. which established that GenAI performed very well at lower level of Bloom's taxonomy with "create" being weakest level of performance. Following this article there are various publications, which have shown GenAI performance in creation of assessment tool and support of students in formative assessment involving learning outcomes with lower level of Bloom's taxonomy. 

 We  understand the concern of authors about students losing critical thinking ability and thereby not being able to complete the learning outcomes. There are ample literature available presently related to this aspect of GenAI assisted assessment. It was expected that the authors would cite suitable references to establish the necessity of this research.

 Methods:

 The authors highlighted five-step method to evaluate and address the impact of GenAI. These five steps are:

 1.Pre-scan 

 2. Quick scan

 3. Detailed scan

 4. Curriculum adaptation

 5. Conslodation

 Let us look at the terminology used in describing the steps and whether prospective reader would be able to undestand the framework with this terminology. 

 Pre-scan step describes evaluation of educational formats before starting the scanning of assessments.

 Quick Scan step describes identification of at risk assessments where use of GenAI remains undetected.

 Detailed scan describes the trial by the lecturers to test whether the assessment can be completed effectively with the help of GenAI. Five rating scales have been developed to classify the effectiveness of GenAI to support the assessments. Curriculum adaptation step helps to categorize learning outcomes and related skills. 

 In contrast to clear description of first four steps, the description of fifth step consolidation is sketchy. 

 The authors have mentioned that the proposed method and case-study offer a potential blueprint for educational institutions to follow. A perfect blue print is transparent, best represented in a tabular manner with steps starting from objectives and ending with steps of implementation with descriptions of steps, allowing easy implemntation. 

 Results

 A birds-eye view of results is clearly depicted using Figure 2 where no. of assessments involved in each step is described in a flow chart.

 However, a list of learning outcomes written in text will allow the prospective reader to understand about the nature of outcome and relationship with the use of GenAI and its effect. Although Figure 4 has described the outcomes progress as per taxonomy. 

 Discussion and Conclusion

 Compared to Introduction, this chapter has been written with a lot of clarity with sufficient evidence of literature. The authors described the process of adoption of GenAI in trasnformation of teaching process.

 Impact: This article is an excellent fit for new educational method. With a few changes the article can be used as a guide for GenAI impact assessment.

Is the rationale for developing the new method (or application) clearly explained?

Partly

Is the description of the method technically sound?

Yes

Are the conclusions about the method and its performance adequately supported by the findings presented in the article?

Yes

If any results are presented, are all the source data underlying the results available to ensure full reproducibility?

Yes

Are sufficient details provided to allow replication of the method development and its use by others?

Partly

Reviewer Expertise:

Technology enhanced learning, AI, Small group Learning, Online assessment

I confirm that I have read this submission and believe that I have an appropriate level of expertise to confirm that it is of an acceptable scientific standard, however I have significant reservations, as outlined above.

# Reviewer report: Reviewer response for version 1

**DOI:** 10.21956/mep.22282.r42321

**Published:** 2025-08-14

**Authors:** Suriyaarachchige Nishan Silva

**Affiliations:** 1 CHI Health, Claremont, Australia; 2 Fellowship Support Program, Royal Australian College of General Practitioners, East Melbourne, Victoria, Australia

It would have been better to elaborate on the five step method to evaluate and address the impact of GenAI. How did you arrive at the 57% of assessments were at risk of reduced validity and relevance?  It is not clear the assessment format mentioned in the second para of results is the same that was adopted by 64%. How was this decided?

Is the rationale for developing the new method (or application) clearly explained?

Partly

Is the description of the method technically sound?

No

Are the conclusions about the method and its performance adequately supported by the findings presented in the article?

Partly

If any results are presented, are all the source data underlying the results available to ensure full reproducibility?

Partly

Are sufficient details provided to allow replication of the method development and its use by others?

No

Reviewer Expertise:

Health Informatician, Health professionals educationist

I confirm that I have read this submission and believe that I have an appropriate level of expertise to confirm that it is of an acceptable scientific standard, however I have significant reservations, as outlined above.

# Response

**Published:** 2026-01-21

**Authors:** Remco Jongkind

**Affiliations:** Teaching & Learning Centre, Amsterdam UMC Locatie AMC, Amsterdam, North Holland, The Netherlands

Dear Suriyaarachchige,

 Thank you for your useful feedback. We elaborated more on the five step method to improve clarity in the method section.

 Regarding the 57%, we included a clarification on page 21 on how we came to the 57%. Namely 55 out of 96 assessments were at risk.

 Furthermore on page 22 we included reasons for the choices to change assessment format or learning goal.

 Lastly we improved the overall language quality of the manuscript for better flow and clarity. Among others to ensure the numerous percentages regarding assessments at risk and lecturer decisions are now more transparent and easier to interpret within the narrative.

 Best wishes,

 Remco

# Reviewer report: Reviewer response for version 1

**DOI:** 10.21956/mep.22282.r41411

**Published:** 2025-05-02

**Authors:** Vijay Rajput, Mohammadali Shoja

**Affiliations:** 1 Nova Southeastern University, Fort Lauderdale, USA; 2 Medical Education, Nova Southeastern University Dr Kiran C Patel College of Allopathic Medicine (Ringgold ID: 503842), Fort Lauderdale, Florida, USA

We read with great interest the manuscript by Jongkind and colleagues, which explores a timely and highly relevant issue: the impact of generative AI on the validity of student assessment and the objectivity of learning outcomes. The authors propose a structured strategy for evaluating how generative AI intersects with learning objectives and assessment design across a curriculum. This is an important and forward-thinking endeavor, particularly given the rapid integration of AI technologies in academic settings.

 That said, we found that the overall language quality of the manuscript could be significantly strengthened to better support its strong conceptual contributions. At present, the writing lacks fluency in places, and some word choices are imprecise, which at times makes it difficult to fully grasp the intended meaning. For example, we occasionally needed to reread sentences to discern their context, and in some cases, the phrasing led to unintended interpretations. One such instance appears in the section titled “Step 1: Pre-scan,” where the authors write, “… it is estimated to which degree generative AI can be used to make these assessments.” This wording might unintentionally suggest that educators are using generative AI to construct assessments, rather than assessing the extent to which students might use AI to complete them—presumably the intended meaning. Similar issues appear throughout the paper and affect the overall clarity and accessibility of the work.

 Nonetheless, the substance of the study is both thoughtful and commendable. The authors present a compelling and well-structured framework for tackling the pedagogical challenges posed by generative AI in education. Their suggestion that learning outcomes may need to be assessed through more interactive or supervised formats is reasonable and well-supported. However, it may be helpful to more explicitly acknowledge the distinctive educational value of traditional written assessments—particularly those completed over time—which can foster critical thinking, reflection, and synthesis in ways that are not always replicable in supervised environments.

 In addition, the manuscript might benefit from addressing the growing importance of generative AI literacy among students. As educational institutions adapt to this new landscape, it will be essential to provide clear guidance on when and how AI tools can be ethically and effectively used. If restrictions are placed on AI usage in certain contexts, what enforcement mechanisms are available? These practical considerations would enhance the applicability of the proposed framework.

 Lastly, it may be helpful to distinguish more clearly between different types of AI use. While employing generative AI to produce ideas or content in place of a student’s own work raises valid concerns about academic integrity, using AI tools to refine language, improve grammar, or enhance structure may be more analogous to the support offered by a writing center or copyeditor—particularly for students writing in a second language. A nuanced discussion of these differences would enrich the paper and help inform more equitable and thoughtful policy development.

 In summary, this manuscript takes on a pressing and important challenge in higher education and offers a promising framework for addressing it. With careful revision to improve the clarity of the language and a more detailed consideration of implementation and ethical nuances, the paper could make a valuable contribution to the evolving conversation around generative AI in education.

Is the rationale for developing the new method (or application) clearly explained?

Partly

Is the description of the method technically sound?

Yes

Are the conclusions about the method and its performance adequately supported by the findings presented in the article?

Yes

If any results are presented, are all the source data underlying the results available to ensure full reproducibility?

Yes

Are sufficient details provided to allow replication of the method development and its use by others?

Yes

Reviewer Expertise:

Medical Education, AI in education, ethics, professionalism

We confirm that we have read this submission and believe that we have an appropriate level of expertise to confirm that it is of an acceptable scientific standard.

# Response

**Published:** 2026-01-21

**Authors:** Remco Jongkind

**Affiliations:** Teaching & Learning Centre, Amsterdam UMC Locatie AMC, Amsterdam, North Holland, The Netherlands

Dear Vijay ****and Mohammadali,

 Thank you for your elaborate and useful feedback!

 Based on your mentioned points of improvement we have implemented several enhancements. Below we provide details per point of improvement mentioned.  

**Overall language quality of the manuscript**

 The overall language quality has been refined for better flow, improving also the sentence mentioned in step 1. Furthermore we ensured the numerous percentages regarding assessments at risk and lecturer decisions are now more transparent and easier to interpret within the narrative.

**Distinctive educational value of traditional written assessments**

 We have more explicitly discussed the inherent educational value of traditional written assessments. Drawing on pedagogical literature, we discuss on page 29 that the process of writing serves as a critical "meaning-making" exercise that fosters deep learning and individual creativity—qualities that risk being bypassed if outsourced to GenAI.

**Importance of generative AI literacy among students**.

 The manuscript now elaborates more on the importance of AI literacy when allowing GenAI use for learning goals. We explain, a.o. on page 23 that if GenAI is permitted, it must be accompanied by explicit instruction on its limitations and ethical use.

 Enforcement mechanisms If restrictions are placed on AI usage in certain contexts

 We have provided more detail on potential alternative assessment formats (e.g. on page 24). We describe specific strategies, such as in-class reflections, oral examinations, and process-oriented grading, designed to ensure that students cannot use GenAI to circumvent achieving learning goals.

**Distinguishing between types of GenAI use for learning goals**

 On page 26-27 we have introduced a clearer distinction between using GenAI for linguistic refinement (e.g., grammar and flow) versus content generation (e.g., idea synthesis and creative output).

 Best wishes,

 Remco Jongkind
